# Investigating Useful Properties of Four *Streptomyces* Strains Active against *Fusarium graminearum* Growth and Deoxynivalenol Production on Wheat Grains by qPCR

**DOI:** 10.3390/toxins12090560

**Published:** 2020-08-31

**Authors:** Elena Maria Colombo, Andrea Kunova, Claudio Gardana, Cristina Pizzatti, Paolo Simonetti, Paolo Cortesi, Marco Saracchi, Matias Pasquali

**Affiliations:** Department of Food, Environmental and Nutritional Science (DeFENS), University of Milan, 20133 Milan, Italy; elenamaria.colombo@unimi.it (E.M.C.); claudio.gardana@unimi.it (C.G.); cristina.pizzatti@unimi.it (C.P.); paolo.simonetti@unimi.it (P.S.); paolo.cortesi@unimi.it (P.C.); marco.saracchi@unimi.it (M.S.); matias.pasquali@unimi.it (M.P.)

**Keywords:** biocontrol, mycotoxin, *Triticum aestivum*, qPCR, ergosterol

## Abstract

*Streptomyces* spp. can be exploited as biocontrol agents (BCAs) against plant pathogens such as *Fusarium graminearum*, the main causal agent of Fusarium head blight (FHB) and against the contamination of grains with deoxynivalenol (DON). In the present research, four *Streptomyces* strains active against *F. graminearum* in dual plate assays were characterized for their ability to colonize detached wheat grains in the presence of *F. graminearum* and to limit DON production. The pathogen and BCA abundance were assessed by a quantitative real-time PCR, while DON production was assessed by HPLC quantification and compared to ergosterol to correlate the toxin production to the amount of fungal mycelium. Fungal growth and mycotoxin production were assessed with both co-inoculation and late inoculation of the BCAs in vitro (three days post-*Fusarium* inoculation) to test the interaction between the fungus and the bacteria. The level of inhibition of the pathogen and the toxin production were strain-specific. Overall, a higher level of DON inhibition (up to 99%) and a strong reduction in fungal biomass (up to 71%) were achieved when streptomycetes were co-inoculated with the fungus. This research enabled studying the antifungal efficacy of the four *Streptomyces* strains and monitoring their development in DON-inducing conditions.

## 1. Introduction

Fusarium head blight (FHB) is one of the most devastating cereal diseases, especially for wheat and barley. It is caused by a complex group of *Fusarium* spp., in which *Fusarium graminearum* is the main causal agent [1,2]. Severe yield losses occur in the field, together with a variable level of grain contamination with mycotoxins belonging to the group of type B trichothecenes, such as deoxynivalenol (DON) [3]. The fungal contamination in harvested grains can be kept under control during the storage period, but the mycotoxin incidence in feed and food products often increases dramatically, becoming a threat for food and feed safety [4,5]. The risks to human and animal health have forced organizations worldwide to establish standards for maximum allowable levels in products for human consumption [6]. The toxic effects of DON include the alteration of intestinal, nervous, and immune systems due to the inhibition of protein synthesis and induction of apoptosis [7].

Several control strategies to manage FHB incidence and consequent DON contamination in harvested grains have been exploited in the field, such as the development of resistant varieties, application of fungicides, and crop rotation [8]. Nevertheless, some agricultural practices can promote *Fusarium* development or enhance mycotoxin accumulation under favorable environmental conditions [9]. As integrative approaches to manage FHB are urgently needed, research on biocontrol agents (BCA) has been receiving increased attention [10]. Biological control is an environmentally friendly approach to fight plant pathogens using microbial antagonists. The complex epidemiology of FHB enables applying antagonists to seeds, crop residues, or to the spikes [11], as well as during post-harvest [12].

*Streptomyces* spp. are Gram-positive bacteria belonging to the phylum *Actinobacteria*, ubiquitous in soil, and commonly exploited for antibiotic production in human and veterinary medicine. They grow through a combination of tip extension and branching of hyphae, forming a vegetative mycelium. Later, in response to nutrient depletion and other signals, they form aerial mycelium-carrying spores [13,14]. In correspondence with these morphological changes, they produce a variety of secondary metabolites active against possible competitors present in their niche [15]. Recently, *Streptomyces* have also been found to establish symbiotic interactions with plants and other eukaryotes [16]. Therefore, they have the potential to become key players for developing novel strategies against plant pathogens and to limit toxin contamination thanks to the discovery of promising metabolites for crop protection [17]. Indeed, recent studies confirmed their ability to reduce toxigenic *Fusarium* spp. growth and disease severity in controlled and field conditions [18,19,20], as well as DON production in vitro and in planta [21,22,23]. Currently, only two commercial products based on live *Streptomyces* spp. are available on the European market (Mycostop^®^ and Actinovate^®^) and neither of them were specifically registered for FHB management [24], despite showing certain activity against *F. graminearum* [25]. However, diverse *Streptomyces* strains are likely to become part of the tools available for integrated disease control in the near future as shown by some recent patent depositions [26].

In an effort to select novel *Streptomyces* strains able to counteract fungal and mycotoxin contamination in a wheat–*Fusarium* pathosystem, their survival on wheat grains and the potential inhibitory effect of a co-culture with the pathogen needs to be assessed. Indeed, biological interactions between *Streptomyces* and *Fusarium* are crucial to understand how possible disease control can be employed and exploited [27].

Ergosterol quantification is routinely used to determine the microbiological status of grains and feeds [28,29,30] and can be exploited to evaluate the effect of a fungicide or a natural product on fungal pathogen growth [31,32]. This fungal marker can also be used to normalize mycotoxin levels based on fungal development [33,34]. Moreover, in recent years, quantitative real-time PCR (qPCR) has been recognized as a rapid and highly sensitive technique to accurately quantify fungal and bacterial biomass in a wide range of food and grain samples [35], a parameter which can be easily correlated with the level of disease observed in the field as well as mycotoxin contamination [36,37]. Additionally, as qPCR is able to detect specific strains, it has been increasingly used for BCA monitoring in the target substrate [38], enabling the assessment of BCA survival under specific abiotic and biotic conditions, as well as the influence of time and application method on its survival [39]. 

In order to study the mechanism of action of four promising *Streptomyces* strains [40,41] against *F. graminearum* growth and toxin production (DON) in vitro, specific qPCR methods were developed to monitor both fungal and BCA strains. These analyses were combined with ergosterol quantification to support the qPCR-based quantification and to normalize the DON content in flour samples. 

The goals of the paper were to test whether in DON-inducing conditions (1) fungal growth is affected by the tested *Streptomyces* strains; (2) toxin production is affected by the tested *Streptomyces* strains; (3) *Streptomyces* strain development is affected by pathogen presence; and (4) the timing of *Streptomyces* inoculation determines their efficacy as BCA in in vitro conditions.

## 2. Results

### 2.1. Streptomyces Influence on Fungal Growth

Four *Streptomyces* strains (DEF09, DEF20, DEF39, and DEF48) were selected on the basis of their promising biocontrol activity against *F. graminearum* [40,41]. The effect of the *Streptomyces* strains on fungal growth on wheat grains was evaluated at two different inoculation times (0 or 3 days post-pathogen inoculation; DPI). *Fusarium graminearum* was quantified by HPLC ergosterol determination and a qPCR based on the *TRI12* gene. Only traces or no detectable levels of ergosterol or qPCR amplifications were detected in blank samples (no-BCA or fungal treatment), confirming that the used seeds were free of *F. graminearum* (Table S1). In addition, the ratios of the fungal amount in the control to that in the treated samples were calculated for both the ergosterol and the qPCR method in order to assess the correlation between the two quantification methods. A correlation value of 0.82 was obtained (Figure S1). 

**Figure 1:**
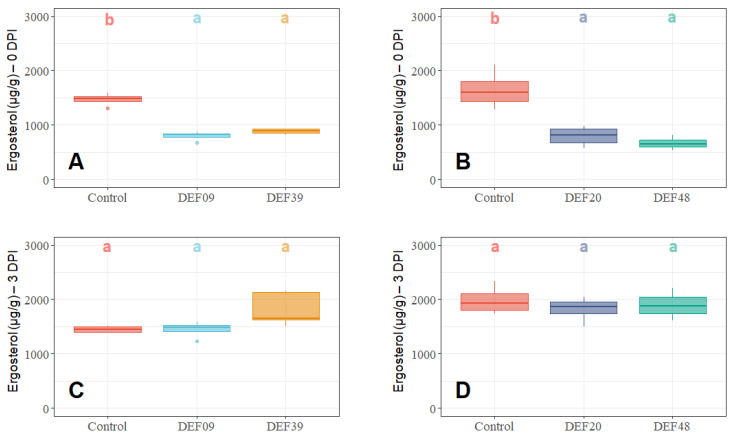
Ergosterol quantification (µg/g) in wheat grain samples treated with *Streptomyces* spp. DEF09, DEF39, DEF20, and DEF48 at 0 days post-inoculation (0 DPI) (**A**,**B**) and 3 DPI (**C**,**D**) after 11 days of incubation. The presence of different control replicates (no-biocontrol agent (BCA) treatment) is due to multiple experiments being performed. Means of four replicates in two technical repetitions were subjected to ANOVA and a post hoc Tukey’s HSD test. Box-plots with the same letters on top of the graph are not significantly different according to Tukey’s HSD test (*p* < 0.05).

**Figure 2:**
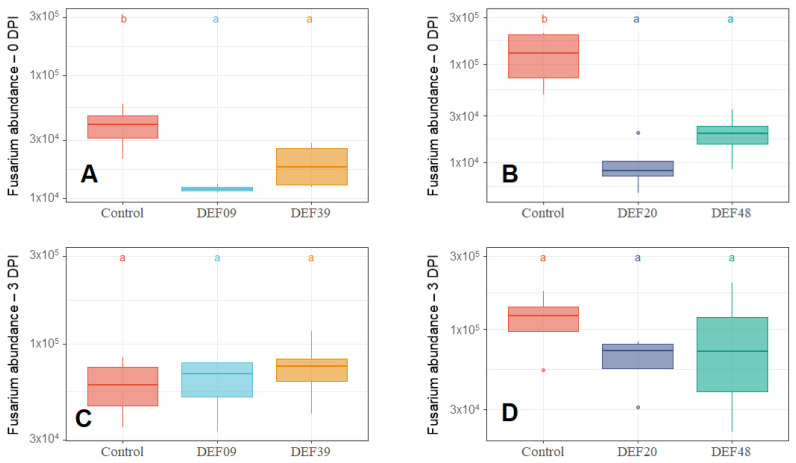
*Fusarium graminearum* CS3005 abundance normalized to wheat abundance in wheat grain samples treated with *Streptomyces* spp. DEF09, DEF39, DEF20, and DEF48 at 0 DPI (**A**,**B**) and 3 DPI (**C**,**D**) after 11 days of incubation. The presence of different control replicates (no-BCA treatment) is due to multiple experiments being performed. Abundance was calculated as a ratio of *Fusarium* DNA to wheat DNA quantity. The means of four replicates were subjected to ANOVA and a post hoc Tukey’s HSD test. Box-plots with the same letters on top of the graph are not significantly different according to Tukey’s HSD test (*p* < 0.05).

When DEF09, DEF20, DEF39, and DEF48 strains were co-cultured with *F. graminearum* from the first day of inoculation (0 DPI), the fungal growth was clearly inhibited by all *Streptomyces* spp. strains (*p* < 0.05) (Figure 1A,B; Figure 2A,B and Figure S2). Instead, the inoculations of DEF09, DEF20, DEF39, and DEF48 strains at three days after fungal inoculation (3 DPI) were conducted to simulate a treatment of an already established *Fusarium* infection, and did not result in any antifungal effect as measured by ergosterol amounts or fungal abundance (Figure 1C,D; Figure 2C,D and Figure S2). All *p*-values from the ANOVA analyses are listed in Table S2. 

The BCAs were able to limit the ergosterol content (*Fusarium* growth) up to 45%, 52%, 40%, and 60% for DEF09, DEF20, DEF39, and DEF48, respectively. *Fusarium* abundance was reduced up to 70%, 92%, 50%, and 85% for DEF09, DEF20, DEF39, and DEF48, respectively. The highest *Fusarium* inhibition was recorded for DEF20 and DEF48 treatments, suggesting they were the most effective strains in limiting *Fusarium* growth, and this conclusion was consistent for both the qPCR and the ergosterol analyses.

### 2.2. Fitness of the Streptomyces Strains on Wheat Grains

**Figure 3:**
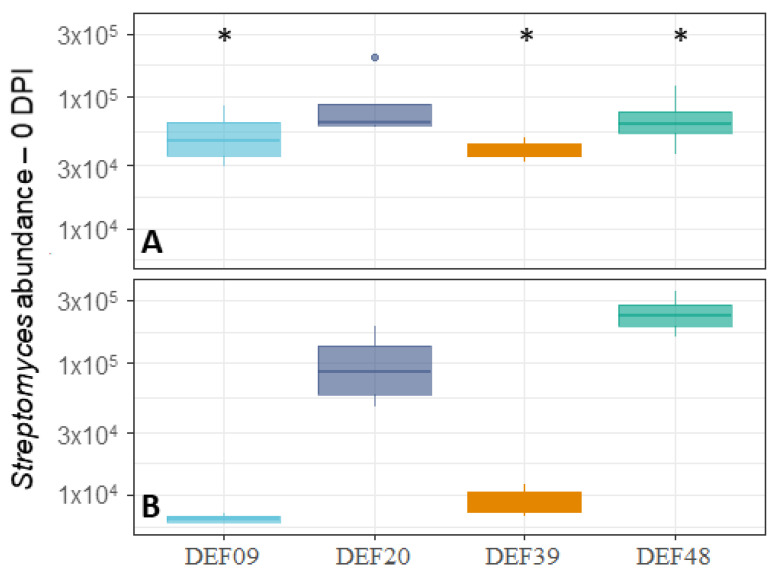
*Streptomyces* spp. strain abundance normalized to wheat abundance in wheat grain samples after 11 days of incubation. * *p* < 0.05 was considered significant in the *t*-test comparison between control replicates (*Streptomyces* alone; (**A**)) and 0 DPI treatment (*Streptomyces* strain + *F. graminearum* CS3005; (**B**)) abundance was calculated as a ratio of *Streptomyces* DNA to wheat DNA quantity.

To assess the fitness of the four *Streptomyces* spp. strains used in this study, their abundance was evaluated both on control- and *Fusarium*-contaminated wheat grains using a qPCR analysis targeting the *Streptymyces recA* gene region. Although the abundance of the four *Streptomyces* spp. was variable on control wheat seeds (Figure 3A), these differences were not statistically significant. Instead, the development of the majority of *Streptomyces* strains was clearly influenced by the presence of the pathogen on *Fusarium*-contaminated wheat grains (Figure 3B). The *t*-test comparison highlighted the differences among BCA development in co-culture with CS3005 or when cultured alone. In particular, DEF48 showed significantly increased growth (*p* = 0.03), while DEF09 and DEF39 were clearly inhibited by the presence of the fungus (*p* = 0.03 and *p* = 0.00). Despite their reduced growth, they were still able to exert antifungal activity against the pathogen (Figure 1A and Figure 2A). DEF20 was not influenced by the co-culture with *F. graminearum* CS3005 (*p* = 0.87). Overall, DEF20 and DEF48 demonstrated a great ability to colonize the substrate and to exhibit a strong antifungal effect in the tested conditions.

### 2.3. Influence of the BCA Treatment on Mycotoxin Production on Detached Wheat Grains 

**Figure 4:**
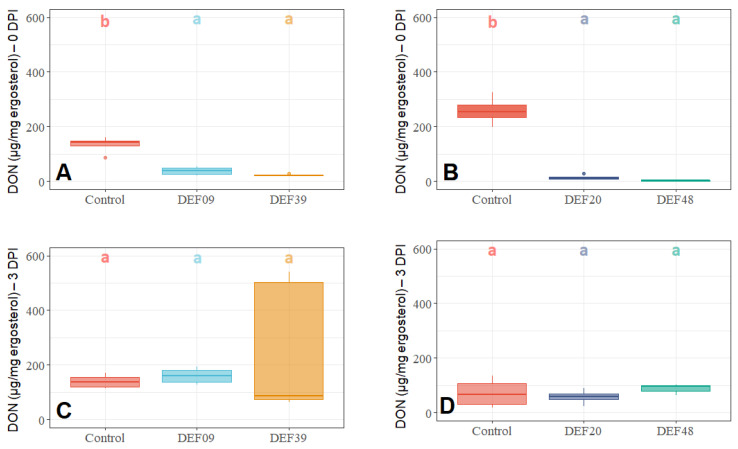
Deoxynivalenol (DON) quantification (µg/mg ergosterol) in wheat grain samples treated with *Streptomyces* spp. DEF09, DEF39, DEF20, and DEF48 at 0 DPI (**A**,**B**) and 3 DPI (**C**,**D**) treatment after 11 days of incubation. The presence of different control replicates (no-BCA treatment) is due to multiple experiments being performed. Means of four replicates were subjected to ANOVA and a post hoc Tukey’s HSD test. Box-plots with the same letters on top of the graph are not significantly different according to Tukey’s HSD test (*p* < 0.05).

To verify the ability of the biocontrol *Streptomyces* spp. strains to limit toxin production, DON amounts were expressed as µg/mg of ergosterol to normalize mycotoxin production to fungal growth [42]. Similarly to what was observed for fungal quantification (Figure 1 and Figure 2), when co-cultured (0 DPI), all four BCAs significantly reduced mycotoxin production (Figure 4A,B). Instead, no significant decrease in toxin amount was recorded when streptomycetes were applied three days after pathogen inoculation (3 DPI, Figure 4C,D).

All strains were able to effectively reduce DON production (µg/mg ergosterol) at 0 DPI treatment according to the following percentages: 71%, 94%, 83%, and 99% for DEF09, DEF20, DEF39, and DEF48, respectively.

## 3. Discussion

Our study contributes to deciphering the interactions occurring between *Streptomyces* strains and a toxigenic strain of *F. graminearum* in a controlled environment, attempting a first characterization of four promising *Streptomyces* strains to be used as BCAs (Table 1). Direct antifungal activity was observed only when the bacteria and the fungus were co-inoculated (0 DPI treatment), confirming that early streptomycete inoculation is a prerequisite for their use as BCAs. These results have important implications on the development of BCAs effective in controlling mycotoxin accumulation in grains.

The fact that the antimycotoxigenic activity is most probably a consequence of the antifungal activity suggests that the BCA needs to colonize the seed before the fungus becomes established, and no curative effects can be expected as confirmed by the absence of the inhibition of fungal growth and mycotoxin production following the treatment at 3 DPI. This might be linked to the *Streptomyces* developmental program, which requires colonization of the substrate with the formation of a vegetative mycelium, and the aerial mycelium with spore production and the synthesis of bioactive compounds occur only in response to nutrient depletion and other signals [14]. Our results are in accordance with previous observations where a higher mycotoxin reduction was obtained after treating peanut grains with a *Streptomyces* strain in a preventive way (24 h before pathogen inoculation) [43]. The appropriate strategy of streptomycete application can substantially improve their efficacy under complex environmental conditions; indeed, several reports have highlighted the importance of allowing the *Streptomyces* spp. to get pre-established in the host tissue/substrate [24,44].

The four studied strains showed different development at 0 DPI. Although DEF09 and DEF39 showed reduced growth in the presence of *Fusarium* in comparison with the control without fungal inoculation, they were still able to reduce mycotoxin production and fungal growth. DEF20 growth was not affected by co-inoculation with the pathogen. Surprisingly, DEF48 biomass increased in response to co-culture with CS3005, and moreover, it showed the highest inhibition of ergosterol and mycotoxin levels, confirming previous reports on the variety of interactions between fusaria and *Streptomyces* [20]. Indeed, some strains can also benefit from the production of fungal metabolites [45,46] and, during this interaction, the production of antifungal metabolites can be elicited [47,48,49]. Therefore, further studies are needed on the interaction with multiple fungi causing DON accumulation [50] to decipher the specific mechanism of activity and the reliability of the BCA in the environment.

A key aspect in the selection of BCAs against toxigenic *Fusarium* spp. is the evaluation of their ability to counteract mycotoxin production [51]. Indeed, the reduction in pathogen development does not always correspond to a reduction in toxin level [52,53]. In the present research, treated samples at 0 DPI not only had inhibited *Fusarium* growth but also reduced mycotoxin levels in comparison to control treatments. A strong level of DON inhibition (µg/mg ergosterol) of above 94% was recorded for DEF20 and DEF48 in parallel with a strong reduction in fungal biomass. High chitinase activity was described for these two strains in a previous study [41], but we cannot exclude contemporary production of other bioactive metabolites able to suppress pathogen development, a common trait characterizing the *Streptomyces* genus [54]. Interestingly, DEF39 showed a remarkable decrease in toxin production (83%) and the lowest inhibition of fungal biomass (40% of ergosterol inhibition). Chitinase activity was weak for this strain [41], suggesting that within its arsenal of secondary metabolites it also harbors the potential to regulate toxin production via specific mechanisms that are not linked to antibiosis alone. Several microbial metabolites have been characterized for their specific inhibitory activity against aflatoxins produced by *Aspergillus parasiticus*, such as dioctatin A, blasticidin A, and aflastatin A [55,56,57]. Future studies will characterize the metabolites of the strain DEF39 to decipher their bioactivity.

In our study, we characterize the behavior of four promising novel BCAs against toxigenic *F. graminearum*, analyzing their ability to inhibit both fungal growth and DON production on wheat grains. As reported in Table 1, our results identify peculiar characteristics of the four analyzed strains: DEF48 has the highest capacity to inhibit fungal growth, DEF20 growth is not influenced by fungal presence, and DEF39 is able to limit DON production in an effective manner despite the low fungal growth inhibition. The panel included also DEF09 that showed very effective inhibition of fungal growth and disease establishment when interacting with the plant in field conditions [41]. In the present study, the DEF09 strain showed lower but significant inhibition of fungal growth and toxin production, confirming that mechanisms of fungal growth inhibition are active in this strain also on detached grains. Our study identifies peculiarities of each strain that can be exploited for generating complex consortia able to effectively limit the disease [58,59]. Hopefully, future integration of omics analyses of the biocontrol strains may lead to the identification of effective molecules able to block fungal growth and modulate toxin biosynthesis [60] as well as to the understanding of mechanisms that modulate growth by sensing the fungal neighbor. A promising approach to developing novel targeted strategies to limit the damage of toxin accumulation in food and feed can come from the integration of this knowledge [61].

## 4. Materials and Methods 

### 4.1. Microorganisms 

The four *Streptomyces* strains (DEF09, DEF20, DEF39, and DEF48) used in this work were part of a collection of isolates maintained in the laboratory of Plant Pathology at the Department of Food, Environmental and Nutritional Sciences (DeFENS), University of Milan, Italy. They were originally isolated from the inner root tissues of graminaceous plants [62]: DEF09 from wheat, DEF20 from *Carex* sp., DEF39 from rye, and DEF48 from corn [40]. These strains showed promising biocontrol features in vitro and in planta against *F. graminearum* in previous works, being able to limit the growth of various *Fusarium* strains in dual culture by over 40% [40,41]. They were grown on Czapek yeast extract medium (CZY: 35 g/L Czapek dox broth, Difco Laboratories, Detroit, MI, USA; 2 g/L yeast extract, Difco Laboratories, Detroit, MI, USA; 15 g/L agar; Amresco Inc., Solon, OH, USA) for 14 days at 24 °C. Spores were collected by adding 5 mL of 10% sterile glycerol (ICN Biomedicals, Irvine, CA, USA) + 0.01% Tween 20 solution (Sigma-Aldrich, St. Louis, MO, USA) to the plate and scraping the surface of the colonies with a sterile loop. The concentration was determined using a haemocytometer and adjusted to 2 × 10^7^ spores/mL. Small aliquots were then stored at −20 °C. 

The toxigenic *Fusarium* strain used in this study was *F. graminearum* CS3005 [63]. The strain was grown on V8 medium (200 mL/L V8 juice, Campbell Soup Company, Camden, NJ, USA; 2 g/L CaCO_3_, Sigma-Aldrich, St. Louis, MO, USA; 15 g/L Agar, Amresco Inc., Solon, OH, USA) for five days at 24 °C. 

### 4.2. BCA treatments on Wheat Grains

Wheat grains of *Triticum aestivum* “Bandera” (20 g) were placed in 100 mL flasks, soaked with 20 mL of deionized water, and autoclaved for 20 min at 120 °C. The treatments consisted of four *Streptomyces* strains, one type of fungus inoculation (*F. graminearum* CS3005), and two different times of antagonist inoculation. 

Grains were treated with 500 µL of *Streptomyces* strain spore suspension (2 × 10^7^ spore/mL) and six agar-mycelium plugs (6 mm in diameter) taken from a *Fusarium* colony. BCAs were applied as follows: 0 DPI (*Streptomyces* spp. were applied at the same time as pathogen inoculation); 3 DPI (*Streptomyces* spp. were applied 3 days post-pathogen inoculation). The time point of 3 days post-inoculation was selected as this corresponds with the onset of toxin synthesis by the fungus [64]. Each combination of treatments was repeated four times. Three controls were included. Blank samples (grain without any treatment) inoculated only with 500 µL of 10% sterile glycerol were prepared to define the background levels for each quantification, the *Streptomyces*-control, in which 500 µL of *Streptomyces* spore suspension (2 × 10^7^ spore/mL) was inoculated, and the *Fusarium* control, in which six agar-mycelium plugs of *F. graminearum* CS3005 were added to the sterilized grains. The incubation was performed at 24 °C for 11 days in the dark. Flasks were monitored and shaken daily, using a spatula when needed. Wheat grains were lyophilized (model Heto-EPD3, Thermo Scientific, San Jose, CA) for 24 h and ground to a fine powder. Samples were kept at −80 °C for subsequent extraction.

Two methods for fungal quantification were evaluated (ergosterol and qPCR). Ergosterol concentration was evaluated by chemical extraction and HPLC analysis (Section 4.4 and Section 4.5). The qPCR-based *Fusarium* quantification was carried out based on the *TRI12* gene (Section 4.6, Section 4.7, Section 4.8).

### 4.3. Chemicals

Standards of ergosterol and deoxynivalenol (DON) were purchased from Sigma-Aldrich (St. Louis, MO, USA). Solvents were purchased from Sigma-Aldrich unless otherwise stated (St. Louis, MO, USA). Water was supplied by a Milli-Q apparatus (Millipore, Milford, MA, USA).

### 4.4. Ergosterol Extraction and Determination 

Ergosterol extraction was performed following the procedure described in a previous research [26]. Briefly, samples were prepared as follows: 400 mg of flour was weighed and extracted overnight using 10 mL of a CHCl_3_/MeOH (2:1 (*v/v*)) solution. After centrifugation at 9880× *g* for 10 min, the supernatant was collected, and the pellet was extracted again with 5 mL of the same solvent solution. The two extracts were combined, and the volume was made up to 20 mL using CHCl_3_/MeOH (2:1 (*v/v*)). The resulting solution was diluted 1:2 prior to injection and chromatographic analysis. Ergosterol levels were used to normalize DON content per fungal mass.

The used HPLC system was an Alliance 2695 (Waters, Milford, MA, USA) equipped with a model 2998 photodiode array detector (Waters, Milford, MA, USA). A 5 µm Hypersil C18 column (250 × 4.6 mm, Thermo Scientific, San Jose, CA, USA) maintained at 30 °C carried out the separation in isocratic mode. The flow rate was 1.0 mL/min, and the eluent was methanol. Samples were maintained at 20 °C. Chromatographic data were acquired from 195 to 350 nm and integrated at 282 nm. Ergosterol stock solution (0.26 mg/mL) was prepared in MeOH and stored at −20 °C. Working solutions (*n* = 7) were prepared in the range of 1.3–130 µg/mL, and 50 µL was injected into the chromatographic system. Each analysis was carried out in duplicate. Ergosterol LOD was 0.2 µg/mL and LOQ was 1.3 µg/mL.

### 4.5. DON Extraction and Determination

To determine the amount of DON, the flour (1 g) was extracted with 10 mL of a water/CH_3_CN (20:80 (*v/v*)) solution under sonication for 30 min. Then, the mixture was centrifuged at 1600× *g* for 10 min, and the supernatant was transferred into a 10 mL flask where the volumes were adjusted using a water/CH_3_CN (20:80 (*v/v*)) solution. The residues were extracted again as described above, and the two extracts were analyzed separately. Mycotoxin determination was carried out using a UHPLC model Acquity (Waters, Milford, MA, USA) coupled with an HR Fourier transform Orbitrap mass spectrometer (model Exactive, Thermo Scientific, San Jose, CA, USA), equipped with a HESI-II probe for ESI and a collision cell (HCD). A Hypersil Gold C_18_ column (100 × 2.1 mm, 1.9 µm, Thermo Scientific, San Jose, CA, USA) was used for the separation. The MS data were processed using Xcalibur software, version 3.0.63 (Thermo Scientific, San Jose, CA, USA). The used operative conditions and the elution gradient have been previously described [40]. DON LOD was determined at 5 ng/mL and LOQ at 50 ng/mL. 

### 4.6. DNA Extraction

The total DNA from flour samples was extracted using a DNA extraction kit (DNeasy *mericon* Food Kit, Qiagen, Hilden, Germany). Briefly, 100 mg of the sample was weighed and processed following the manufacturer’s instructions with one minor modification whereby 5 µL of 20 mg/mL RNase A (Invitrogen, Thermo Fisher Scientific, Waltham, MA, USA) was added during the first incubation step at 60 °C. Quantification and verification of the 260/280 ratio of the extracted DNA were carried out with a Take3 Micro-Volume plate in a microplate reader (Synergy H1, Biotek, Winooski, VT, USA). Spectrophotometric quantification was confirmed by fluorometric quantification. DNA degradation level was assessed on electrophoretic gel. DNA samples were stored in Elution buffer (EB) (Qiagen, Hilden, Germany) supplied by the DNA extraction kit at 4 °C.

### 4.7. Primers and PCR Analysis

Details of the primers used in this study are listed in Table 2. In order to obtain *recA* gene sequences of *Streptomyces* strains involved in this study, DNA was extracted as previously described [65]. Briefly, a small amount of *Streptomyces* spores and mycelium was transferred to a sterile microcentrifuge tube containing 27 µL Tris (10 mM)-EDTA (1 mM) (pH 7.6) with a sterile toothpick; then, 3 µL 0.4 M KOH-10 mM EDTA was added to the tube and incubated at 70 °C for 5 min. Next, 3 µL Tris-HCl (pH 4.0) was added to the lysate to adjust the pH. The lysate was used directly as a DNA template for PCR amplification using primers *recA*PF and *recA*PR [66]. PCR was performed in a total volume of 25 µL, which contained 0.25 µL of GoTaq^®^ DNA Polymerase 5 U/µL (Promega, Madison, WI, USA), 5 µL of Green GoTaq^®^ Reaction Buffer 5× (Promega, Madison, WI, USA), 1 µL of 10 mM dNTP (Promega, Madison, WI, USA), 1 µL of 10 mM forward primer, 1 µL of 10 mM reverse primer, and 1 µL of template DNA in nuclease-free water. The reaction conditions were initial denaturation at 95 °C for 5 min, followed by 35 cycles of denaturation at 95 °C for 20 s, annealing at 60 °C for 30 s, and extension at 72 °C for 90 s. A final extension was performed at 72 °C for 7 min. Reaction products were separated by electrophoresis on a 1% agarose gel containing ethidium bromide and visualized under UV light. The PCR products were sequenced (Eurofins Genomics, Ebersberg, Germany) using the *recA*F primer [66]. Specific primers were designed for *Streptomyces* quantification based on *recA* gene polymorphisms. Sequences of the *recA* gene of DEF09, DEF20, DEF39, and DEF48 (deposited in NCBI with accession numbers MN207071-MN207074) were aligned using Geneious software (Biomatters, Auckland, New Zealand), version R11.1.4 (Figure S3). Primers for *F. graminearum* quantification were designed based on the *TRI12* gene sequence (NC_026475). In both cases, primer specificity was tested using NCBI Primer-BLAST with default parameters against the nr database to assess the level of specificity and identify potential mistargets. Bioinformatic analysis confirmed that the specificity of the *recA* primers is limited to *Streptomyces* spp. (File S1). Similarly, a novel primer set was designed based on a specific region of *TRI12* which proved species specificity to *Fusarium* spp. (File S2). To verify the absence of unspecific amplification of newly designed primers with other targets in wheat grains, DNA of untreated grains was amplified with *recA* and *TRI12* primers. No signals above the LOQ were detected.

Hor1f and Hor2r primers [67], based on the plant *EF1α* gene, were used as an internal control for each sample and to normalize fungal and bacterial DNA quantities. *Streptomyces* spp. and *F. graminearum* quantities were calculated as the target DNA/wheat DNA molecule number using the formula described in File S3 [68]. 

### 4.8. qPCR Analysis

Quantitative real-time PCR (qPCR) was performed in order to evaluate the amount of *Fusarium*, *Streptomyces*, and wheat DNA in milled grain samples following The Minimum Information for Publication of Quantitative Real-Time PCR Experiments (MIQE) guidelines [69]. Prior to experimentation, the primers, PCR protocol specifications, and thermocycling parameters were adapted to the available reaction mixes and laboratory devices. All sample dilutions were prepared simultaneously using DEPC-treated and nuclease-free water (Fischer Scientific, Thermo Fisher Scientific, Waltham, MA, USA), and stored dilutions were kept at 4 °C during the week of the experiments. Primers were stored in aliquots at −20 °C. 

In addition, the possible interference of wheat DNA in *Streptomyces* and *Fusarium* quantification was checked by adding 5 ng of wheat DNA extracted from untreated samples as previously described. Three replicates of each dilution (5, 0.5, 0.05, 0.005, and 0.0005 ng) of the fungal and bacterial DNA were prepared and used to build the respective standard curves. Two non-template controls (NTCs) were used: water-only and background DNA-only samples. The reaction efficiency and determination coefficient (R^2^) were calculated based on the obtained Cq values. As there was no influence on Cq values due to the presence of wheat DNA (data not shown), all the experiments were carried out with standard curves that were freshly prepared with DEPC-treated and nuclease-free water (Fisher Scientific, Thermo Fisher Scientific, Waltham, MA, USA).

The qPCR reactions were carried out using an Applied Biosystems QuantStudio 3 PCR System (Thermo Fisher Scientific, Waltham, MA, USA) in standard mode. The amplification mix consisted of 2× PowerSYBR Green PCR Master Mix (Applied Biosystems, Thermo Fisher Scientific, Waltham, MA, USA), 0.3 μM of each primer, and 5 μL template DNA (1 ng/μL) in DEPC-treated and nuclease-free water (Fisher Scientific, Thermo Fisher Scientific, Waltham, MA, USA) in a total volume of 20 μL. The adopted amplification protocol was 2 min at 50 °C, 10 min at 95 °C, followed by 40 cycles with 15 s at 95 °C for denaturation and 60 s at 60 °C for primer annealing, extension, and data collection. After the amplification reaction, melting curve analysis was used to determine the specificity of the amplification products by incubating them for 15 s at 95 °C and 60 s at 60 °C and then reading the fluorescence at 0.15 °C increments from 60 to 95 °C. Primer specificity was verified by the presence of a single peak in the melting curve. Analysis of each DNA target (*Fusarium*, *Streptomyces*, or wheat) was conducted in 96-well optical reaction plates (Applied Biosystems, Thermo Fisher Scientific, Waltham, MA, USA) covered with Microamp optical adhesive film (Applied Biosystems, Thermo Fisher Scientific, Waltham, MA, USA), and included a standard curve for quantification that was prepared for each pair of primers with *F. graminearum* PH1 genomic DNA [70], *Streptomyces* spp. DEF09 genomic DNA, and wheat genomic DNA. The first concentration of the standard curve was 10 ng, then six dilutions 1:10 (5 ng to 0.00005 ng) were selected based on previous experiments. Three replicates were performed to check the linearity of the assay and efficiencies were calculated for each plate. Each sample was amplified in triplicate and NTCs were added to each plate in triplicate.

Cq values were obtained by using QuantStudio™ Design and Analysis Software version 1.5.0 (Thermo Fisher Scientific, Waltham, MA, USA) and exporting the amplification results into Excel format. The threshold for the Cq analysis was manually adjusted for each amplified target. Outliers among the three technical replicates were discarded if the standard error of Cq was higher than 0.2. PCR efficiency was calculated from standard curves using the formula based on regression slopes, where the efficiency (E) of the different assays was E = 10^−1/slope^. Normalization of the curves was obtained using the standard curves obtained for each plate as calibrator. All of the amplification results and calculations are available in File S3. Based on standard curves, the LOQ for *recA* and *TRI12* assays were established to be 0.05 and 5 pg, respectively.

The amount of fungal, bacterial, and plant DNA was calculated using the adjusted Cq and the selected standard curve. After the transformation of the DNA quantity in DNA molecule numbers, the *Streptomyces*, *Fusarium*, and wheat abundance was calculated based on the size of each genome (see File S3), *Streptomyces* and *Fusarium* abundances were normalized with the wheat DNA abundance for each sample. 

### 4.9. Data Analysis

All the statistical analyses were performed using R software, version R 3.5.3 [71], unless otherwise stated. *p* < 0.05 was considered significant.

The whole study was divided into multiple experiments in order to manage the analysis. Therefore, each experimental set required separate control replicates (no-BCA treatment).

The efficacy of the bacterial antagonist treatment against fungal growth and DON production was assessed as the difference between controls and treated samples. Therefore, ergosterol quantification data, *Fusarium* abundance (expressed as *Fusarium*/wheat DNA molecule number), and DON level (expressed as µg DON/mg ergosterol) were subjected to ANOVA followed by a Tukey HSD post hoc test for multiple comparison. 

In addition, the ratio between fungal quantity in control samples and treated samples was evaluated for ergosterol and qPCR data. In particular, from the qPCR data for each sample, the average of the ng of fungal DNA in control samples was divided by the ng of fungal DNA in the treated samples. Ergosterol ratios were calculated as the average of the µg of ergosterol in control treatments divided by the µg of ergosterol in the treated samples. The correlation between the ergosterol and qPCR ratios was then estimated. 

In order to understand the influence of *Fusarium* presence on the development, the growth of *Streptomyces* strains at 0 DPI treatment was evaluated, comparing *Streptomyces* abundance in control samples (no-*Fusarium* spp.) and those obtained from co-cultured samples (*Fusarium* spp. + *Streptomyces* spp.). Data were subject to comparison using a *t*-test. 

## Figures and Tables

**Table 1:** Strain ID, isolation source of the endophytic strains, in vitro inhibition of *F. graminearum* growth in dual plate assays, protection against Fusarium foot rot (FFR) symptoms on wheat plants in greenhouses, effect on *F. graminearum* growth on wheat grains, inhibition of DON production, and possible mechanism of reduction of toxin accumulation by different strains. The underlined data are previously published results [40,41].

Strain	Isolation Source	Fungal Growth Inhibition in vitro [40,41]	FFR Reduction on Plants (Greenhouse) [40,41]	Inhibition of *F. graminearum* Mycelium Growth on Grains at 0DPI		Toxin Inhibition in Grains Infected by *F. graminearum* 0 DPI	*Streptomyces* Growth in Grains Infected by *F. graminearum* 0 DPI	Possible Mechanism of DON Reduction
				qPCR	Ergosterol			
DEF09	*Triticum aestivum*	59%	46%	70%	45%	71%	-	Mostly mycelium growth reduction
DEF20	*Carex* sp.	78%	11%	92%	52%	94%	=	Mostly mycelium growth reduction
DEF39	*Secale cereale*	64%	0%	50%	40%	83%	-	Specific toxin inhibition independent from inhibition of mycelium growth
EF48	*Zea mays*	70%	29%	85%	60%	99%	+	Mostly mycelium growth reduction

Table footer: ”-“ reduced growth compared to control, “=” equal growth compared to control, “+” increased growth compared to control.

**Table 2:** Primers used in the study for the amplification and sequencing of the *recA* gene together with those used to quantify the three targets (*Streptomyces* spp., *F. graminearum*, and wheat).

Primer Name	Assay Target	Primer Sequence	Melting Temp.	GC %	Reference
*recA*PF	*Streptomyces* spp.	CCGCRCTCGCACAGATTGAACGSCAATTC	70.2	56.9	[56]
*recA*PR	*Streptomyces* spp.	GCSAGGTCGGGGTTGTCCTTSAGGAAGTTGCG	74.6	56.9	[56]
*recA*F	*Streptomyces* spp.	ACAGATTGAACGGCAATTCG	55.3	45	[56]
*recA*R	*Streptomyces* spp.	ACCTTGTTCTTGACCACCTT	55.3	45	[56]
qstreptoREcAF	*Streptomyces* spp.	AAGATCACCAGTGCGCTCAA	59.96	50	This study
qstreptoREcAR	*Streptomyces* spp.	GAGCTGGTTGATGAAGATCGC	59.40	52	This study
TRI12QF	*F. graminearum*	ATCTCAGCCAGACGACAGGT	59.87	55	This study
TRI12DR	*F. graminearum*	CGAGGCGAGGTGTAATATCC	59.55	55	This study
Hor1f	Wheat	TCTCTGGGTTTGAGGGTGAC	62	55	[57]
Hor2r	Wheat	GGCCCTTGTACCAGTCAAGGT	51	57	[57]

## Supplementary Materials

The following are available online at https://www.mdpi.com/2072-6651/12/9/560/s1, Figure S1: Scatter plot of ratios calculated between total ng of fungal DNA in control and treated samples and ratios of ergosterol (µg/g) in control and treated samples, Figure S2: *Fusarium graminearum* growth in the flasks on wheat grains after co-inoculation (0 DPI) and late inoculation (3 DPI) with *Streptomyces* spp. strains DEF09, DEF20, DEF39 and DEF 48 after 11 days of incubation, Figure S3: Position of qstreptoREcAF and qstreptoREcAR on *recA* alignment obtained from the four *Streptomyces* strains involved in this study, Table S1: *TRI12* (ng) and ergosterol quantification in blank samples (no-*Fusarium* or *Streptomyces* inoculation), Table S2: *p*-values of ANOVA analyses, comparing the effects of BCA treatments on fungal biomass detection by the two analysis methods (ergosterol and qPCR). In addition, results from Tukey’s HSD post hoc comparison are listed as significant difference (*p* < 0.05) of the amount of ergosterol or fungal abundance in comparison with the untreated control (no-*Streptomyces* inoculation), File S1: Products of target templates (qstreptoREcAF/qstreptoREcAF) using NCBI Primer-BLAST (default parameters, nr database), File S2: Products on target templates (TRI12QF/TRI12DR) using NCBI Primer-BLAST (default parameters, nr database), File S3: Raw data and additional information for qPCR analysis including MIQE checklist.
