# Supplementary material for: Investigating Useful Properties of Four Streptomyces Strains Active against Fusarium graminearum Growth and Deoxynivalenol Production on Wheat Grains by qPCR
Source: Toxins (Basel). 2020 Aug 31;12(9):560. doi: 10.3390/toxins12090560 (PMC7551252; doi:10.3390/toxins12090560)
Supplement: Supplementary file 1 [file toxins-12-00560-s001.zip › toxins-877381-Supplementary files/Figure S3.pdf]

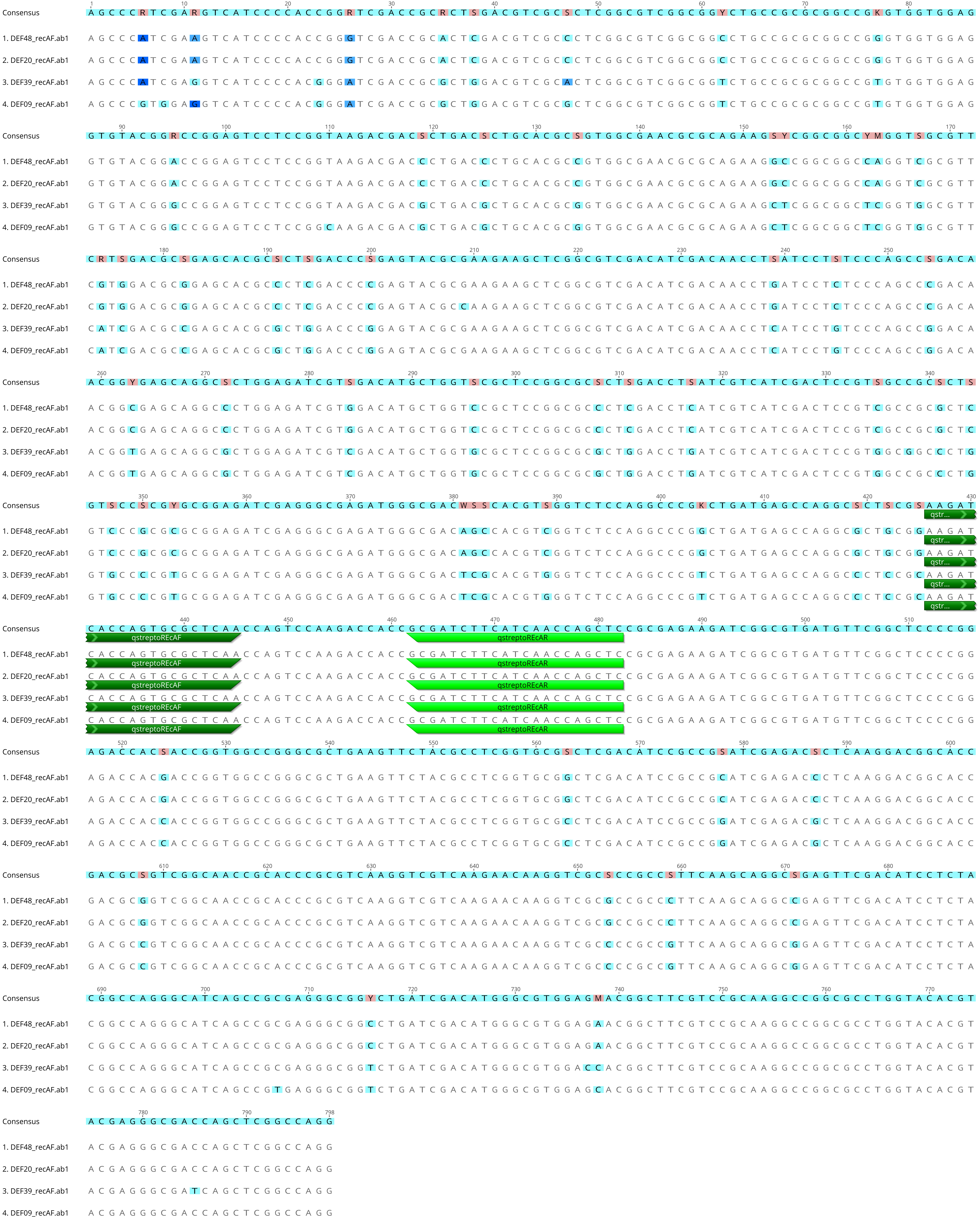
Consensus
1. DEF48_recAF.ab1
2. DEF20_recAF.ab1
3. DEF39_recAF.ab1
4. DEF09_recAF.ab1
qstreptoRECAF
qstreptoRECAR
