# Supplementary material for: Investigating Useful Properties of Four Streptomyces Strains Active against Fusarium graminearum Growth and Deoxynivalenol Production on Wheat Grains by qPCR
Source: Toxins (Basel). 2020 Aug 31;12(9):560. doi: 10.3390/toxins12090560 (PMC7551252; doi:10.3390/toxins12090560)
Supplement: Supplementary file 1 [file toxins-12-00560-s001.zip › toxins-877381-Supplementary files/File S1.docx]

**PRODUCTS ON TARGET TEMPLATES (qstreptoREcAF/qstreptoREcAF) USING PRIMER BLAST (DEFAULT PARAMETERS, NR DATABASE)**

>[CP034687.1](https://www.ncbi.nlm.nih.gov/nucleotide/1547209052?from=2536484&to=2536540&report=gbwithparts) Streptomyces griseoviridis strain F1-27 chromosome, complete genome

product length = 57

Features associated with this product:

[recombinase RecA](https://www.ncbi.nlm.nih.gov/nucleotide/1547209052?from=2535950&to=2537068&report=gbwithparts)

Forward primer 1 AAGATCACCAGTGCGCTCAA 20

Template 2536540 .................... 2536521

Reverse primer 1 GAGCTGGTTGATGAAGATCGC 21

Template 2536484 ..................... 2536504

>[CP029601.1](https://www.ncbi.nlm.nih.gov/nucleotide/1535781911?from=2109404&to=2109460&report=gbwithparts) Streptomyces sp. WAC 01438 chromosome, complete genome

product length = 57

Features associated with this product:

[recombinase RecA](https://www.ncbi.nlm.nih.gov/nucleotide/1535781911?from=2108873&to=2109988&report=gbwithparts)

Forward primer 1 AAGATCACCAGTGCGCTCAA 20

Template 2109460 .................... 2109441

Reverse primer 1 GAGCTGGTTGATGAAGATCGC 21

Template 2109404 ..................... 2109424

>[CP029361.1](https://www.ncbi.nlm.nih.gov/nucleotide/1390670665?from=1582491&to=1582547&report=gbwithparts) Streptomyces globisporus strain TFH56 chromosome, complete genome

product length = 57

Features associated with this product:

[recombinase RecA](https://www.ncbi.nlm.nih.gov/nucleotide/1390670665?from=1581951&to=1583075&report=gbwithparts)

Forward primer 1 AAGATCACCAGTGCGCTCAA 20

Template 1582547 .................... 1582528

Reverse primer 1 GAGCTGGTTGATGAAGATCGC 21

Template 1582491 ..................... 1582511

>[CP021707.1](https://www.ncbi.nlm.nih.gov/nucleotide/1304301697?from=5882168&to=5882224&report=gbwithparts) Streptomyces sp. S063, complete genome

product length = 57

Forward primer 1 AAGATCACCAGTGCGCTCAA 20

Template 5882168 .................... 5882187

Reverse primer 1 GAGCTGGTTGATGAAGATCGC 21

Template 5882224 ..................... 5882204

>[MF455355.1](https://www.ncbi.nlm.nih.gov/entrez/viewer.fcgi?db=nucleotide&id=1284806567) Streptomyces sp. CB02613 recombinase A (recA) gene, partial cds

product length = 57

Forward primer 1 AAGATCACCAGTGCGCTCAA 20

Template 460 .................... 479

Reverse primer 1 GAGCTGGTTGATGAAGATCGC 21

Template 516 ..................... 496

>[CP021748.1](https://www.ncbi.nlm.nih.gov/nucleotide/1205255223?from=6971790&to=6971846&report=gbwithparts) Streptomyces alboflavus strain MDJK44, complete genome

product length = 57

Features associated with this product:

[recombinase RecA](https://www.ncbi.nlm.nih.gov/nucleotide/1205255223?from=6971262&to=6972389&report=gbwithparts)

Forward primer 1 AAGATCACCAGTGCGCTCAA 20

Template 6971790 .................... 6971809

Reverse primer 1 GAGCTGGTTGATGAAGATCGC 21

Template 6971846 ..................... 6971826

>[KX582226.1](https://www.ncbi.nlm.nih.gov/entrez/viewer.fcgi?db=nucleotide&id=1198363698) Streptomyces sp. MOLA 1615 recombinase A (recA) gene, partial cds

product length = 57

Forward primer 1 AAGATCACCAGTGCGCTCAA 20

Template 385 .................... 404

Reverse primer 1 GAGCTGGTTGATGAAGATCGC 21

Template 441 ..................... 421

>[KX582225.1](https://www.ncbi.nlm.nih.gov/entrez/viewer.fcgi?db=nucleotide&id=1198363696) Streptomyces sp. MOLA 1612 recombinase A (recA) gene, partial cds

product length = 57

Forward primer 1 AAGATCACCAGTGCGCTCAA 20

Template 385 .................... 404

Reverse primer 1 GAGCTGGTTGATGAAGATCGC 21

Template 441 ..................... 421

>[KX582224.1](https://www.ncbi.nlm.nih.gov/entrez/viewer.fcgi?db=nucleotide&id=1198363694) Streptomyces sp. MOLA 1611 recombinase A (recA) gene, partial cds

product length = 57

Forward primer 1 AAGATCACCAGTGCGCTCAA 20

Template 385 .................... 404

Reverse primer 1 GAGCTGGTTGATGAAGATCGC 21

Template 441 ..................... 421

>[KX582223.1](https://www.ncbi.nlm.nih.gov/entrez/viewer.fcgi?db=nucleotide&id=1198363692) Streptomyces sp. MOLA 1596 recombinase A (recA) gene, partial cds

product length = 57

Forward primer 1 AAGATCACCAGTGCGCTCAA 20

Template 385 .................... 404

Reverse primer 1 GAGCTGGTTGATGAAGATCGC 21

Template 441 ..................... 421

>[KX582222.1](https://www.ncbi.nlm.nih.gov/entrez/viewer.fcgi?db=nucleotide&id=1198363690) Streptomyces sp. MOLA 1578 recombinase A (recA) gene, partial cds

product length = 57

Forward primer 1 AAGATCACCAGTGCGCTCAA 20

Template 385 .................... 404

Reverse primer 1 GAGCTGGTTGATGAAGATCGC 21

Template 441 ..................... 421

>[KX582218.1](https://www.ncbi.nlm.nih.gov/entrez/viewer.fcgi?db=nucleotide&id=1198363682) Streptomyces sp. MOLA1421 recombinase A (recA) gene, partial cds

product length = 57

Forward primer 1 AAGATCACCAGTGCGCTCAA 20

Template 385 .................... 404

Reverse primer 1 GAGCTGGTTGATGAAGATCGC 21

Template 441 ..................... 421

>[KX582217.1](https://www.ncbi.nlm.nih.gov/entrez/viewer.fcgi?db=nucleotide&id=1198363680) Streptomyces sp. MOLA 1420 recombinase A (recA) gene, partial cds

product length = 57

Forward primer 1 AAGATCACCAGTGCGCTCAA 20

Template 385 .................... 404

Reverse primer 1 GAGCTGGTTGATGAAGATCGC 21

Template 441 ..................... 421

>[CP020570.1](https://www.ncbi.nlm.nih.gov/nucleotide/1175647368?from=6239839&to=6239895&report=gbwithparts) Streptomyces violaceoruber strain S21, complete genome

product length = 57

Forward primer 1 AAGATCACCAGTGCGCTCAA 20

Template 6239839 .................... 6239858

Reverse primer 1 GAGCTGGTTGATGAAGATCGC 21

Template 6239895 ..................... 6239875

>[KX503536.1](https://www.ncbi.nlm.nih.gov/entrez/viewer.fcgi?db=nucleotide&id=1104598988) Streptomyces sp. strain MM87 RecA (recA) gene, partial cds

product length = 57

Forward primer 1 AAGATCACCAGTGCGCTCAA 20

Template 529 .................... 548

Reverse primer 1 GAGCTGGTTGATGAAGATCGC 21

Template 585 ..................... 565

>[KX503518.1](https://www.ncbi.nlm.nih.gov/entrez/viewer.fcgi?db=nucleotide&id=1104598952) Streptomyces sp. strain MM46 RecA (recA) gene, partial cds

product length = 57

Forward primer 1 AAGATCACCAGTGCGCTCAA 20

Template 529 .................... 548

Reverse primer 1 GAGCTGGTTGATGAAGATCGC 21

Template 585 ..................... 565

>[KX503491.1](https://www.ncbi.nlm.nih.gov/entrez/viewer.fcgi?db=nucleotide&id=1104598898) Streptomyces sp. strain MM12 RecA (recA) gene, partial cds

product length = 57

Forward primer 1 AAGATCACCAGTGCGCTCAA 20

Template 529 .................... 548

Reverse primer 1 GAGCTGGTTGATGAAGATCGC 21

Template 585 ..................... 565

>[KX503488.1](https://www.ncbi.nlm.nih.gov/entrez/viewer.fcgi?db=nucleotide&id=1104598892) Streptomyces sp. strain MM122 RecA (recA) gene, partial cds

product length = 57

Forward primer 1 AAGATCACCAGTGCGCTCAA 20

Template 529 .................... 548

Reverse primer 1 GAGCTGGTTGATGAAGATCGC 21

Template 585 ..................... 565

>[KX503477.1](https://www.ncbi.nlm.nih.gov/entrez/viewer.fcgi?db=nucleotide&id=1104598870) Streptomyces sp. strain MM106 RecA (recA) gene, partial cds

product length = 57

Forward primer 1 AAGATCACCAGTGCGCTCAA 20

Template 529 .................... 548

Reverse primer 1 GAGCTGGTTGATGAAGATCGC 21

Template 585 ..................... 565

>[KT385491.1](https://www.ncbi.nlm.nih.gov/entrez/viewer.fcgi?db=nucleotide&id=1051345875) Streptomyces rhizophilus strain NRRL B-59132 recombinase A (recA) gene, partial cds

product length = 57

Forward primer 1 AAGATCACCAGTGCGCTCAA 20

Template 388 .................... 407

Reverse primer 1 GAGCTGGTTGATGAAGATCGC 21

Template 444 ..................... 424

>[KT385484.1](https://www.ncbi.nlm.nih.gov/entrez/viewer.fcgi?db=nucleotide&id=1051345861) Streptomyces cocklensis strain NRRL B-24911 recombinase A (recA) gene, partial cds

product length = 57

Forward primer 1 AAGATCACCAGTGCGCTCAA 20

Template 388 .................... 407

Reverse primer 1 GAGCTGGTTGATGAAGATCGC 21

Template 444 ..................... 424

>[KT385424.1](https://www.ncbi.nlm.nih.gov/entrez/viewer.fcgi?db=nucleotide&id=1051345741) Streptomyces somaliensis strain NRRL B-12077 recombinase A (recA) gene, partial cds

product length = 57

Forward primer 1 AAGATCACCAGTGCGCTCAA 20

Template 388 .................... 407

Reverse primer 1 GAGCTGGTTGATGAAGATCGC 21

Template 444 ..................... 424

>[KT385414.1](https://www.ncbi.nlm.nih.gov/entrez/viewer.fcgi?db=nucleotide&id=1051345721) Streptomyces rubidus strain NRRL B-24619 recombinase A (recA) gene, partial cds

product length = 57

Forward primer 1 AAGATCACCAGTGCGCTCAA 20

Template 388 .................... 407

Reverse primer 1 GAGCTGGTTGATGAAGATCGC 21

Template 444 ..................... 424

>[KT385396.1](https://www.ncbi.nlm.nih.gov/entrez/viewer.fcgi?db=nucleotide&id=1051345685) Streptomyces pseudovenezuelae strain NRRL B-3623 recombinase A (recA) gene, partial cds

product length = 57

Forward primer 1 AAGATCACCAGTGCGCTCAA 20

Template 388 .................... 407

Reverse primer 1 GAGCTGGTTGATGAAGATCGC 21

Template 444 ..................... 424

>[KT385376.1](https://www.ncbi.nlm.nih.gov/entrez/viewer.fcgi?db=nucleotide&id=1051345645) Streptomyces parvisporogenes strain NRRL B-5464 recombinase A (recA) gene, partial cds

product length = 57

Forward primer 1 AAGATCACCAGTGCGCTCAA 20

Template 388 .................... 407

Reverse primer 1 GAGCTGGTTGATGAAGATCGC 21

Template 444 ..................... 424

>[KT385358.1](https://www.ncbi.nlm.nih.gov/entrez/viewer.fcgi?db=nucleotide&id=1051345609) Streptomyces netropsis strain NRRL ISP-5259 recombinase A (recA) gene, partial cds

product length = 57

Forward primer 1 AAGATCACCAGTGCGCTCAA 20

Template 388 .................... 407

Reverse primer 1 GAGCTGGTTGATGAAGATCGC 21

Template 444 ..................... 424

>[KT385327.1](https://www.ncbi.nlm.nih.gov/entrez/viewer.fcgi?db=nucleotide&id=1051345547) Streptomyces longispororuber strain NRRL B-3736 recombinase A (recA) gene, partial cds

product length = 57

Forward primer 1 AAGATCACCAGTGCGCTCAA 20

Template 388 .................... 407

Reverse primer 1 GAGCTGGTTGATGAAGATCGC 21

Template 444 ..................... 424

>[KT385315.1](https://www.ncbi.nlm.nih.gov/entrez/viewer.fcgi?db=nucleotide&id=1051345523) Streptomyces lateritius strain NRRL B-5349 recombinase A (recA) gene, partial cds

product length = 57

Forward primer 1 AAGATCACCAGTGCGCTCAA 20

Template 388 .................... 407

Reverse primer 1 GAGCTGGTTGATGAAGATCGC 21

Template 444 ..................... 424

>[KT385310.1](https://www.ncbi.nlm.nih.gov/entrez/viewer.fcgi?db=nucleotide&id=1051345513) Streptomyces kunmingensis strain NRRL B-16240 recombinase A (recA) gene, partial cds

product length = 57

Forward primer 1 AAGATCACCAGTGCGCTCAA 20

Template 388 .................... 407

Reverse primer 1 GAGCTGGTTGATGAAGATCGC 21

Template 444 ..................... 424

>[KT385266.1](https://www.ncbi.nlm.nih.gov/entrez/viewer.fcgi?db=nucleotide&id=1051345425) Streptomyces globisporus subsp. globisporus strain NRRL B-2872 recombinase A (recA) gene, partial cds

product length = 57

Forward primer 1 AAGATCACCAGTGCGCTCAA 20

Template 388 .................... 407

Reverse primer 1 GAGCTGGTTGATGAAGATCGC 21

Template 444 ..................... 424

>[KT385207.1](https://www.ncbi.nlm.nih.gov/entrez/viewer.fcgi?db=nucleotide&id=1051345307) Streptomyces anulatus strain NRRL 2250 recombinase A (recA) gene, partial cds

product length = 57

Forward primer 1 AAGATCACCAGTGCGCTCAA 20

Template 388 .................... 407

Reverse primer 1 GAGCTGGTTGATGAAGATCGC 21

Template 444 ..................... 424

>[KT385195.1](https://www.ncbi.nlm.nih.gov/entrez/viewer.fcgi?db=nucleotide&id=1051345283) Streptomyces canarius strain NRRL ISP-5528 recombinase A (recA) gene, partial cds

product length = 57

Forward primer 1 AAGATCACCAGTGCGCTCAA 20

Template 388 .................... 407

Reverse primer 1 GAGCTGGTTGATGAAGATCGC 21

Template 444 ..................... 424

>[KY407306.1](https://www.ncbi.nlm.nih.gov/entrez/viewer.fcgi?db=nucleotide&id=1127931863) Streptomyces sp. strain AC569 recombinase A (recA) gene, partial cds

product length = 57

Forward primer 1 AAGATCACCAGTGCGCTCAA 20

Template 388 .................... 407

Reverse primer 1 GAGCTGGTTGATGAAGATCGC 21

Template 444 ..................... 424

>[KY407302.1](https://www.ncbi.nlm.nih.gov/entrez/viewer.fcgi?db=nucleotide&id=1127931855) Streptomyces sp. strain AC555 recombinase A (recA) gene, partial cds

product length = 57

Forward primer 1 AAGATCACCAGTGCGCTCAA 20

Template 388 .................... 407

Reverse primer 1 GAGCTGGTTGATGAAGATCGC 21

Template 444 ..................... 424

>[KY407286.1](https://www.ncbi.nlm.nih.gov/entrez/viewer.fcgi?db=nucleotide&id=1127931823) Streptomyces sp. strain AC52 recombinase A (recA) gene, partial cds

product length = 57

Forward primer 1 AAGATCACCAGTGCGCTCAA 20

Template 388 .................... 407

Reverse primer 1 GAGCTGGTTGATGAAGATCGC 21

Template 444 ..................... 424

>[CP010833.1](https://www.ncbi.nlm.nih.gov/nucleotide/1127396112?from=1808713&to=1808769&report=gbwithparts) Streptomyces sp. Tue6075, complete genome

product length = 57

Forward primer 1 AAGATCACCAGTGCGCTCAA 20

Template 1808769 .................... 1808750

Reverse primer 1 GAGCTGGTTGATGAAGATCGC 21

Template 1808713 ..................... 1808733

>[KX110980.1](https://www.ncbi.nlm.nih.gov/entrez/viewer.fcgi?db=nucleotide&id=1040180538) Streptomyces sp. st196 recombinase A (recA) gene, partial cds

product length = 57

Forward primer 1 AAGATCACCAGTGCGCTCAA 20

Template 377 .................... 396

Reverse primer 1 GAGCTGGTTGATGAAGATCGC 21

Template 433 ..................... 413

>[KX110979.1](https://www.ncbi.nlm.nih.gov/entrez/viewer.fcgi?db=nucleotide&id=1040180536) Streptomyces sp. st140 recombinase A (recA) gene, partial cds

product length = 57

Forward primer 1 AAGATCACCAGTGCGCTCAA 20

Template 377 .................... 396

Reverse primer 1 GAGCTGGTTGATGAAGATCGC 21

Template 433 ..................... 413

>[KX110978.1](https://www.ncbi.nlm.nih.gov/entrez/viewer.fcgi?db=nucleotide&id=1040180534) Streptomyces sp. f150 recombinase A (recA) gene, partial cds

product length = 57

Forward primer 1 AAGATCACCAGTGCGCTCAA 20

Template 377 .................... 396

Reverse primer 1 GAGCTGGTTGATGAAGATCGC 21

Template 433 ..................... 413

>[KX110977.1](https://www.ncbi.nlm.nih.gov/entrez/viewer.fcgi?db=nucleotide&id=1040180532) Streptomyces sp. ms181 recombinase A (recA) gene, partial cds

product length = 57

Forward primer 1 AAGATCACCAGTGCGCTCAA 20

Template 377 .................... 396

Reverse primer 1 GAGCTGGTTGATGAAGATCGC 21

Template 433 ..................... 413

>[KX110976.1](https://www.ncbi.nlm.nih.gov/entrez/viewer.fcgi?db=nucleotide&id=1040180530) Streptomyces sp. ms115 recombinase A (recA) gene, partial cds

product length = 57

Forward primer 1 AAGATCACCAGTGCGCTCAA 20

Template 377 .................... 396

Reverse primer 1 GAGCTGGTTGATGAAGATCGC 21

Template 433 ..................... 413

>[KX110975.1](https://www.ncbi.nlm.nih.gov/entrez/viewer.fcgi?db=nucleotide&id=1040180528) Streptomyces sp. f61 recombinase A (recA) gene, partial cds

product length = 57

Forward primer 1 AAGATCACCAGTGCGCTCAA 20

Template 377 .................... 396

Reverse primer 1 GAGCTGGTTGATGAAGATCGC 21

Template 433 ..................... 413

>[KX110974.1](https://www.ncbi.nlm.nih.gov/entrez/viewer.fcgi?db=nucleotide&id=1040180526) Streptomyces sp. ms152 recombinase A (recA) gene, partial cds

product length = 57

Forward primer 1 AAGATCACCAGTGCGCTCAA 20

Template 377 .................... 396

Reverse primer 1 GAGCTGGTTGATGAAGATCGC 21

Template 433 ..................... 413

>[KX110973.1](https://www.ncbi.nlm.nih.gov/entrez/viewer.fcgi?db=nucleotide&id=1040180524) Streptomyces sp. f252 recombinase A (recA) gene, partial cds

product length = 57

Forward primer 1 AAGATCACCAGTGCGCTCAA 20

Template 377 .................... 396

Reverse primer 1 GAGCTGGTTGATGAAGATCGC 21

Template 433 ..................... 413

>[KX110970.1](https://www.ncbi.nlm.nih.gov/entrez/viewer.fcgi?db=nucleotide&id=1040180518) Streptomyces sp. ms98 recombinase A (recA) gene, partial cds

product length = 57

Forward primer 1 AAGATCACCAGTGCGCTCAA 20

Template 377 .................... 396

Reverse primer 1 GAGCTGGTTGATGAAGATCGC 21

Template 433 ..................... 413

>[KX110968.1](https://www.ncbi.nlm.nih.gov/entrez/viewer.fcgi?db=nucleotide&id=1040180514) Streptomyces sp. gb15 recombinase A (recA) gene, partial cds

product length = 57

Forward primer 1 AAGATCACCAGTGCGCTCAA 20

Template 377 .................... 396

Reverse primer 1 GAGCTGGTTGATGAAGATCGC 21

Template 433 ..................... 413

>[KX110967.1](https://www.ncbi.nlm.nih.gov/entrez/viewer.fcgi?db=nucleotide&id=1040180512) Streptomyces sp. man185 recombinase A (recA) gene, partial cds

product length = 57

Forward primer 1 AAGATCACCAGTGCGCTCAA 20

Template 377 .................... 396

Reverse primer 1 GAGCTGGTTGATGAAGATCGC 21

Template 433 ..................... 413

>[KX110966.1](https://www.ncbi.nlm.nih.gov/entrez/viewer.fcgi?db=nucleotide&id=1040180510) Streptomyces sp. ms183 recombinase A (recA) gene, partial cds

product length = 57

Forward primer 1 AAGATCACCAGTGCGCTCAA 20

Template 377 .................... 396

Reverse primer 1 GAGCTGGTTGATGAAGATCGC 21

Template 433 ..................... 413

>[KX110963.1](https://www.ncbi.nlm.nih.gov/entrez/viewer.fcgi?db=nucleotide&id=1040180504) Streptomyces sp. ms140 recombinase A (recA) gene, partial cds

product length = 57

Forward primer 1 AAGATCACCAGTGCGCTCAA 20

Template 377 .................... 396

Reverse primer 1 GAGCTGGTTGATGAAGATCGC 21

Template 433 ..................... 413

>[KX110952.1](https://www.ncbi.nlm.nih.gov/entrez/viewer.fcgi?db=nucleotide&id=1040180482) Streptomyces sp. b89 recombinase A (recA) gene, partial cds

product length = 57

Forward primer 1 AAGATCACCAGTGCGCTCAA 20

Template 377 .................... 396

Reverse primer 1 GAGCTGGTTGATGAAGATCGC 21

Template 433 ..................... 413

>[KX110951.1](https://www.ncbi.nlm.nih.gov/entrez/viewer.fcgi?db=nucleotide&id=1040180480) Streptomyces sp. b71 recombinase A (recA) gene, partial cds

product length = 57

Forward primer 1 AAGATCACCAGTGCGCTCAA 20

Template 377 .................... 396

Reverse primer 1 GAGCTGGTTGATGAAGATCGC 21

Template 433 ..................... 413

>[KX110945.1](https://www.ncbi.nlm.nih.gov/entrez/viewer.fcgi?db=nucleotide&id=1040180468) Streptomyces sp. b84 recombinase A (recA) gene, partial cds

product length = 57

Forward primer 1 AAGATCACCAGTGCGCTCAA 20

Template 377 .................... 396

Reverse primer 1 GAGCTGGTTGATGAAGATCGC 21

Template 433 ..................... 413

>[KX110894.1](https://www.ncbi.nlm.nih.gov/entrez/viewer.fcgi?db=nucleotide&id=1040180366) Streptomyces sp. sun103 recombinase A (recA) gene, partial cds

product length = 57

Forward primer 1 AAGATCACCAGTGCGCTCAA 20

Template 377 .................... 396

Reverse primer 1 GAGCTGGTTGATGAAGATCGC 21

Template 433 ..................... 413

>[KX110892.1](https://www.ncbi.nlm.nih.gov/entrez/viewer.fcgi?db=nucleotide&id=1040180362) Streptomyces sp. uw7 recombinase A (recA) gene, partial cds

product length = 57

Forward primer 1 AAGATCACCAGTGCGCTCAA 20

Template 377 .................... 396

Reverse primer 1 GAGCTGGTTGATGAAGATCGC 21

Template 433 ..................... 413

>[KX110891.1](https://www.ncbi.nlm.nih.gov/entrez/viewer.fcgi?db=nucleotide&id=1040180360) Streptomyces sp. uw103 recombinase A (recA) gene, partial cds

product length = 57

Forward primer 1 AAGATCACCAGTGCGCTCAA 20

Template 377 .................... 396

Reverse primer 1 GAGCTGGTTGATGAAGATCGC 21

Template 433 ..................... 413

>[KX110890.1](https://www.ncbi.nlm.nih.gov/entrez/viewer.fcgi?db=nucleotide&id=1040180358) Streptomyces sp. f160 recombinase A (recA) gene, partial cds

product length = 57

Forward primer 1 AAGATCACCAGTGCGCTCAA 20

Template 377 .................... 396

Reverse primer 1 GAGCTGGTTGATGAAGATCGC 21

Template 433 ..................... 413

>[KX110889.1](https://www.ncbi.nlm.nih.gov/entrez/viewer.fcgi?db=nucleotide&id=1040180356) Streptomyces sp. uw102 recombinase A (recA) gene, partial cds

product length = 57

Forward primer 1 AAGATCACCAGTGCGCTCAA 20

Template 377 .................... 396

Reverse primer 1 GAGCTGGTTGATGAAGATCGC 21

Template 433 ..................... 413

>[KX110887.1](https://www.ncbi.nlm.nih.gov/entrez/viewer.fcgi?db=nucleotide&id=1040180352) Streptomyces sp. uw104 recombinase A (recA) gene, partial cds

product length = 57

Forward primer 1 AAGATCACCAGTGCGCTCAA 20

Template 377 .................... 396

Reverse primer 1 GAGCTGGTTGATGAAGATCGC 21

Template 433 ..................... 413

>[KX110886.1](https://www.ncbi.nlm.nih.gov/entrez/viewer.fcgi?db=nucleotide&id=1040180350) Streptomyces sp. f121(2016) strain f121 recombinase A (recA) gene, partial cds

product length = 57

Forward primer 1 AAGATCACCAGTGCGCTCAA 20

Template 377 .................... 396

Reverse primer 1 GAGCTGGTTGATGAAGATCGC 21

Template 433 ..................... 413

>[KX110885.1](https://www.ncbi.nlm.nih.gov/entrez/viewer.fcgi?db=nucleotide&id=1040180348) Streptomyces sp. f189 recombinase A (recA) gene, partial cds

product length = 57

Forward primer 1 AAGATCACCAGTGCGCTCAA 20

Template 377 .................... 396

Reverse primer 1 GAGCTGGTTGATGAAGATCGC 21

Template 433 ..................... 413

>[KX110883.1](https://www.ncbi.nlm.nih.gov/entrez/viewer.fcgi?db=nucleotide&id=1040180344) Streptomyces sp. uw2 recombinase A (recA) gene, partial cds

product length = 57

Forward primer 1 AAGATCACCAGTGCGCTCAA 20

Template 377 .................... 396

Reverse primer 1 GAGCTGGTTGATGAAGATCGC 21

Template 433 ..................... 413

>[KX110882.1](https://www.ncbi.nlm.nih.gov/entrez/viewer.fcgi?db=nucleotide&id=1040180342) Streptomyces sp. ms189 recombinase A (recA) gene, partial cds

product length = 57

Forward primer 1 AAGATCACCAGTGCGCTCAA 20

Template 377 .................... 396

Reverse primer 1 GAGCTGGTTGATGAAGATCGC 21

Template 433 ..................... 413

>[KX110872.1](https://www.ncbi.nlm.nih.gov/entrez/viewer.fcgi?db=nucleotide&id=1040180322) Streptomyces sp. f159 recombinase A (recA) gene, partial cds

product length = 57

Forward primer 1 AAGATCACCAGTGCGCTCAA 20

Template 377 .................... 396

Reverse primer 1 GAGCTGGTTGATGAAGATCGC 21

Template 433 ..................... 413

>[KX110871.1](https://www.ncbi.nlm.nih.gov/entrez/viewer.fcgi?db=nucleotide&id=1040180320) Streptomyces sp. f11(2016) strain f11 recombinase A (recA) gene, partial cds

product length = 57

Forward primer 1 AAGATCACCAGTGCGCTCAA 20

Template 377 .................... 396

Reverse primer 1 GAGCTGGTTGATGAAGATCGC 21

Template 433 ..................... 413

>[KX110849.1](https://www.ncbi.nlm.nih.gov/entrez/viewer.fcgi?db=nucleotide&id=1040180276) Streptomyces sp. f120 recombinase A (recA) gene, partial cds

product length = 57

Forward primer 1 AAGATCACCAGTGCGCTCAA 20

Template 377 .................... 396

Reverse primer 1 GAGCTGGTTGATGAAGATCGC 21

Template 433 ..................... 413

>[KT844518.1](https://www.ncbi.nlm.nih.gov/entrez/viewer.fcgi?db=nucleotide&id=987894888) Streptomyces malachitospinus strain DSM 41828 recombinase A (recA) gene, partial cds

product length = 57

Forward primer 1 AAGATCACCAGTGCGCTCAA 20

Template 373 .................... 392

Reverse primer 1 GAGCTGGTTGATGAAGATCGC 21

Template 429 ..................... 409

>[KT844516.1](https://www.ncbi.nlm.nih.gov/entrez/viewer.fcgi?db=nucleotide&id=987894884) Streptomyces coeruleofuscus strain DSM 40144 recombinase A (recA) gene, partial cds

product length = 57

Forward primer 1 AAGATCACCAGTGCGCTCAA 20

Template 373 .................... 392

Reverse primer 1 GAGCTGGTTGATGAAGATCGC 21

Template 429 ..................... 409

>[KU238303.1](https://www.ncbi.nlm.nih.gov/entrez/viewer.fcgi?db=nucleotide&id=987856543) Streptomyces sp. uw7 recombinase A (recA) gene, partial cds

product length = 57

Forward primer 1 AAGATCACCAGTGCGCTCAA 20

Template 388 .................... 407

Reverse primer 1 GAGCTGGTTGATGAAGATCGC 21

Template 444 ..................... 424

>[KU238300.1](https://www.ncbi.nlm.nih.gov/entrez/viewer.fcgi?db=nucleotide&id=987856537) Streptomyces sp. uw2 recombinase A (recA) gene, partial cds

product length = 57

Forward primer 1 AAGATCACCAGTGCGCTCAA 20

Template 388 .................... 407

Reverse primer 1 GAGCTGGTTGATGAAGATCGC 21

Template 444 ..................... 424

>[KU238299.1](https://www.ncbi.nlm.nih.gov/entrez/viewer.fcgi?db=nucleotide&id=987856535) Streptomyces sp. uw104 recombinase A (recA) gene, partial cds

product length = 57

Forward primer 1 AAGATCACCAGTGCGCTCAA 20

Template 388 .................... 407

Reverse primer 1 GAGCTGGTTGATGAAGATCGC 21

Template 444 ..................... 424

>[KU238298.1](https://www.ncbi.nlm.nih.gov/entrez/viewer.fcgi?db=nucleotide&id=987856533) Streptomyces sp. uw103 recombinase A (recA) gene, partial cds

product length = 57

Forward primer 1 AAGATCACCAGTGCGCTCAA 20

Template 388 .................... 407

Reverse primer 1 GAGCTGGTTGATGAAGATCGC 21

Template 444 ..................... 424

>[KU238297.1](https://www.ncbi.nlm.nih.gov/entrez/viewer.fcgi?db=nucleotide&id=987856531) Streptomyces sp. uw102 recombinase A (recA) gene, partial cds

product length = 57

Forward primer 1 AAGATCACCAGTGCGCTCAA 20

Template 388 .................... 407

Reverse primer 1 GAGCTGGTTGATGAAGATCGC 21

Template 444 ..................... 424

>[KU238296.1](https://www.ncbi.nlm.nih.gov/entrez/viewer.fcgi?db=nucleotide&id=987856529) Streptomyces sp. sun93 recombinase A (recA) gene, partial cds

product length = 57

Forward primer 1 AAGATCACCAGTGCGCTCAA 20

Template 388 .................... 407

Reverse primer 1 GAGCTGGTTGATGAAGATCGC 21

Template 444 ..................... 424

>[KU238294.1](https://www.ncbi.nlm.nih.gov/entrez/viewer.fcgi?db=nucleotide&id=987856525) Streptomyces sp. sun103 recombinase A (recA) gene, partial cds

product length = 57

Forward primer 1 AAGATCACCAGTGCGCTCAA 20

Template 388 .................... 407

Reverse primer 1 GAGCTGGTTGATGAAGATCGC 21

Template 444 ..................... 424

>[KU238286.1](https://www.ncbi.nlm.nih.gov/entrez/viewer.fcgi?db=nucleotide&id=987856509) Streptomyces sp. f189 recombinase A (recA) gene, partial cds

product length = 57

Forward primer 1 AAGATCACCAGTGCGCTCAA 20

Template 388 .................... 407

Reverse primer 1 GAGCTGGTTGATGAAGATCGC 21

Template 444 ..................... 424

>[KU238285.1](https://www.ncbi.nlm.nih.gov/entrez/viewer.fcgi?db=nucleotide&id=987856507) Streptomyces sp. f160 recombinase A (recA) gene, partial cds

product length = 57

Forward primer 1 AAGATCACCAGTGCGCTCAA 20

Template 388 .................... 407

Reverse primer 1 GAGCTGGTTGATGAAGATCGC 21

Template 444 ..................... 424

>[KU238284.1](https://www.ncbi.nlm.nih.gov/entrez/viewer.fcgi?db=nucleotide&id=987856505) Streptomyces sp. f159 recombinase A (recA) gene, partial cds

product length = 57

Forward primer 1 AAGATCACCAGTGCGCTCAA 20

Template 388 .................... 407

Reverse primer 1 GAGCTGGTTGATGAAGATCGC 21

Template 444 ..................... 424

>[KU238283.1](https://www.ncbi.nlm.nih.gov/entrez/viewer.fcgi?db=nucleotide&id=987856503) Streptomyces sp. f11(2016) recombinase A (recA) gene, partial cds

product length = 57

Forward primer 1 AAGATCACCAGTGCGCTCAA 20

Template 388 .................... 407

Reverse primer 1 GAGCTGGTTGATGAAGATCGC 21

Template 444 ..................... 424

>[CP013738.1](https://www.ncbi.nlm.nih.gov/nucleotide/971154291?from=6059629&to=6059685&report=gbwithparts) Streptomyces globisporus C-1027, complete genome

product length = 57

Forward primer 1 AAGATCACCAGTGCGCTCAA 20

Template 6059629 .................... 6059648

Reverse primer 1 GAGCTGGTTGATGAAGATCGC 21

Template 6059685 ..................... 6059665

>[KP890259.1](https://www.ncbi.nlm.nih.gov/entrez/viewer.fcgi?db=nucleotide&id=891151414) Streptomyces incanus strain NRRL B-59129 recombinase A (recA) gene, partial cds

product length = 57

Forward primer 1 AAGATCACCAGTGCGCTCAA 20

Template 388 .................... 407

Reverse primer 1 GAGCTGGTTGATGAAGATCGC 21

Template 444 ..................... 424

>[KP890258.1](https://www.ncbi.nlm.nih.gov/entrez/viewer.fcgi?db=nucleotide&id=891151412) Streptomyces herbaceus strain NRRL B-59128 recombinase A (recA) gene, partial cds

product length = 57

Forward primer 1 AAGATCACCAGTGCGCTCAA 20

Template 388 .................... 407

Reverse primer 1 GAGCTGGTTGATGAAGATCGC 21

Template 444 ..................... 424

>[KF981732.1](https://www.ncbi.nlm.nih.gov/entrez/viewer.fcgi?db=nucleotide&id=589230244) Streptomyces griseus strain CB00830 recombinase A (recA) gene, partial cds

product length = 57

Forward primer 1 AAGATCACCAGTGCGCTCAA 20

Template 461 .................... 480

Reverse primer 1 GAGCTGGTTGATGAAGATCGC 21

Template 517 ..................... 497

>[JF424063.1](https://www.ncbi.nlm.nih.gov/entrez/viewer.fcgi?db=nucleotide&id=343457105) Streptomyces sindenensis strain KCTC 19971 recombinase A (recA) gene, partial cds

product length = 57

Forward primer 1 AAGATCACCAGTGCGCTCAA 20

Template 409 .................... 428

Reverse primer 1 GAGCTGGTTGATGAAGATCGC 21

Template 465 ..................... 445

>[JF424062.1](https://www.ncbi.nlm.nih.gov/entrez/viewer.fcgi?db=nucleotide&id=343457103) Streptomyces rubiginosohelvolus strain KCTC 19970 recombinase A (recA) gene, partial cds

product length = 57

Forward primer 1 AAGATCACCAGTGCGCTCAA 20

Template 409 .................... 428

Reverse primer 1 GAGCTGGTTGATGAAGATCGC 21

Template 465 ..................... 445

>[JF424060.1](https://www.ncbi.nlm.nih.gov/entrez/viewer.fcgi?db=nucleotide&id=343457099) Streptomyces praecox strain KCTC 19965 recombinase A (recA) gene, partial cds

product length = 57

Forward primer 1 AAGATCACCAGTGCGCTCAA 20

Template 409 .................... 428

Reverse primer 1 GAGCTGGTTGATGAAGATCGC 21

Template 465 ..................... 445

>[JF424059.1](https://www.ncbi.nlm.nih.gov/entrez/viewer.fcgi?db=nucleotide&id=343457097) Streptomyces parvisporogenes strain KCTC 19964 recombinase A (recA) gene, partial cds

product length = 57

Forward primer 1 AAGATCACCAGTGCGCTCAA 20

Template 409 .................... 428

Reverse primer 1 GAGCTGGTTGATGAAGATCGC 21

Template 465 ..................... 445

>[JF424058.1](https://www.ncbi.nlm.nih.gov/entrez/viewer.fcgi?db=nucleotide&id=343457095) Streptomyces olivaceiscleroticus strain KCTC 19963 recombinase A (recA) gene, partial cds

product length = 57

Forward primer 1 AAGATCACCAGTGCGCTCAA 20

Template 409 .................... 428

Reverse primer 1 GAGCTGGTTGATGAAGATCGC 21

Template 465 ..................... 445

>[JF424053.1](https://www.ncbi.nlm.nih.gov/entrez/viewer.fcgi?db=nucleotide&id=343457085) Streptomyces californicus strain KCTC 19957 recombinase A (recA) gene, partial cds

product length = 57

Forward primer 1 AAGATCACCAGTGCGCTCAA 20

Template 409 .................... 428

Reverse primer 1 GAGCTGGTTGATGAAGATCGC 21

Template 465 ..................... 445

>[JF424052.1](https://www.ncbi.nlm.nih.gov/entrez/viewer.fcgi?db=nucleotide&id=343457083) Streptomyces aureoversilis strain KCTC 19956 recombinase A (recA) gene, partial cds

product length = 57

Forward primer 1 AAGATCACCAGTGCGCTCAA 20

Template 409 .................... 428

Reverse primer 1 GAGCTGGTTGATGAAGATCGC 21

Template 465 ..................... 445

>[JF424051.1](https://www.ncbi.nlm.nih.gov/entrez/viewer.fcgi?db=nucleotide&id=343457081) Streptomyces speibonae strain KCTC 9973 recombinase A (recA) gene, partial cds

product length = 57

Forward primer 1 AAGATCACCAGTGCGCTCAA 20

Template 409 .................... 428

Reverse primer 1 GAGCTGGTTGATGAAGATCGC 21

Template 465 ..................... 445

>[JF424050.1](https://www.ncbi.nlm.nih.gov/entrez/viewer.fcgi?db=nucleotide&id=343457079) Streptomyces netropsis strain KCTC 9873 recombinase A (recA) gene, partial cds

product length = 57

Forward primer 1 AAGATCACCAGTGCGCTCAA 20

Template 409 .................... 428

Reverse primer 1 GAGCTGGTTGATGAAGATCGC 21

Template 465 ..................... 445

>[HQ244484.1](https://www.ncbi.nlm.nih.gov/entrez/viewer.fcgi?db=nucleotide&id=325551429) Streptomyces niger strain CGMCC 4.1748 recombinase A (recA) gene, partial cds

product length = 57

Forward primer 1 AAGATCACCAGTGCGCTCAA 20

Template 388 .................... 407

Reverse primer 1 GAGCTGGTTGATGAAGATCGC 21

Template 444 ..................... 424

>[FJ406280.1](https://www.ncbi.nlm.nih.gov/entrez/viewer.fcgi?db=nucleotide&id=220961049) Streptomyces griseoverticillatus strain DSM 40507 recombinase A (recA) gene, partial cds

product length = 57

Forward primer 1 AAGATCACCAGTGCGCTCAA 20

Template 388 .................... 407

Reverse primer 1 GAGCTGGTTGATGAAGATCGC 21

Template 444 ..................... 424

>[FJ406276.1](https://www.ncbi.nlm.nih.gov/entrez/viewer.fcgi?db=nucleotide&id=220961041) Streptomyces olivaceiscleroticus strain AS 4.1991 recombinase A (recA) gene, partial cds

product length = 57

Forward primer 1 AAGATCACCAGTGCGCTCAA 20

Template 388 .................... 407

Reverse primer 1 GAGCTGGTTGATGAAGATCGC 21

Template 444 ..................... 424

>[FJ406259.1](https://www.ncbi.nlm.nih.gov/entrez/viewer.fcgi?db=nucleotide&id=220961007) Streptomyces aureoversilis strain AS 4.1641 recombinase A (recA) gene, partial cds

product length = 57

Forward primer 1 AAGATCACCAGTGCGCTCAA 20

Template 388 .................... 407

Reverse primer 1 GAGCTGGTTGATGAAGATCGC 21

Template 444 ..................... 424

>[FJ406257.1](https://www.ncbi.nlm.nih.gov/entrez/viewer.fcgi?db=nucleotide&id=220961003) Streptomyces coerulescens strain AS 4.1597 recombinase A (recA) gene, partial cds

product length = 57

Forward primer 1 AAGATCACCAGTGCGCTCAA 20

Template 388 .................... 407

Reverse primer 1 GAGCTGGTTGATGAAGATCGC 21

Template 444 ..................... 424

>[EF055068.1](https://www.ncbi.nlm.nih.gov/entrez/viewer.fcgi?db=nucleotide&id=117667709) Streptomyces fulvorobeus strain JCM 9090 recombinase A (recA) gene, partial cds

product length = 57

Forward primer 1 AAGATCACCAGTGCGCTCAA 20

Template 388 .................... 407

Reverse primer 1 GAGCTGGTTGATGAAGATCGC 21

Template 444 ..................... 424

>[EF055065.1](https://www.ncbi.nlm.nih.gov/entrez/viewer.fcgi?db=nucleotide&id=117667703) Streptomyces pulveraceus strain AS 4.1928 recombinase A (recA) gene, partial cds

product length = 57

Forward primer 1 AAGATCACCAGTGCGCTCAA 20

Template 388 .................... 407

Reverse primer 1 GAGCTGGTTGATGAAGATCGC 21

Template 444 ..................... 424

>[EF055040.1](https://www.ncbi.nlm.nih.gov/entrez/viewer.fcgi?db=nucleotide&id=117667653) Streptomyces albovinaceus strain AS 4.1631 recombinase A (recA) gene, partial cds

product length = 57

Forward primer 1 AAGATCACCAGTGCGCTCAA 20

Template 388 .................... 407

Reverse primer 1 GAGCTGGTTGATGAAGATCGC 21

Template 444 ..................... 424

>[EF055027.1](https://www.ncbi.nlm.nih.gov/entrez/viewer.fcgi?db=nucleotide&id=117667627) Streptomyces anulatus strain AS 4.1421 recombinase A (recA) gene, partial cds

product length = 57

Forward primer 1 AAGATCACCAGTGCGCTCAA 20

Template 388 .................... 407

Reverse primer 1 GAGCTGGTTGATGAAGATCGC 21

Template 444 ..................... 424

>[EF055046.1](https://www.ncbi.nlm.nih.gov/entrez/viewer.fcgi?db=nucleotide&id=117667665) Streptomyces spiroverticillatus strain AS 4.1749 recombinase A (recA) gene, partial cds

product length = 57

Forward primer 1 AAGATCACCAGTGCGCTCAA 20

Template 388 .................... 407

Reverse primer 1 GAGCTGGTTGATGAAGATCGC 21

Template 444 ..................... 424

>[EF055025.1](https://www.ncbi.nlm.nih.gov/entrez/viewer.fcgi?db=nucleotide&id=117667623) Streptomyces exfoliatus strain AS 4.1407 recombinase A (recA) gene, partial cds

product length = 57

Forward primer 1 AAGATCACCAGTGCGCTCAA 20

Template 388 .................... 407

Reverse primer 1 GAGCTGGTTGATGAAGATCGC 21

Template 444 ..................... 424

>[EF055024.1](https://www.ncbi.nlm.nih.gov/entrez/viewer.fcgi?db=nucleotide&id=117667621) Streptomyces badius strain AS 4.1406 recombinase A (recA) gene, partial cds

product length = 57

Forward primer 1 AAGATCACCAGTGCGCTCAA 20

Template 388 .................... 407

Reverse primer 1 GAGCTGGTTGATGAAGATCGC 21

Template 444 ..................... 424

>[EF661762.1](https://www.ncbi.nlm.nih.gov/entrez/viewer.fcgi?db=nucleotide&id=157000372) Streptomyces nitrosporeus strain CGMCC 4.1973 recombinase A (recA) gene, partial cds

product length = 57

Forward primer 1 AAGATCACCAGTGCGCTCAA 20

Template 388 .................... 407

Reverse primer 1 GAGCTGGTTGATGAAGATCGC 21

Template 444 ..................... 424

>[EF055016.1](https://www.ncbi.nlm.nih.gov/entrez/viewer.fcgi?db=nucleotide&id=117667605) Streptomyces sindenensis strain AS 4.626 recombinase A (recA) gene, partial cds

product length = 57

Forward primer 1 AAGATCACCAGTGCGCTCAA 20

Template 388 .................... 407

Reverse primer 1 GAGCTGGTTGATGAAGATCGC 21

Template 444 ..................... 424

>[EF055015.1](https://www.ncbi.nlm.nih.gov/entrez/viewer.fcgi?db=nucleotide&id=117667603) Streptomyces californicus strain AS 4.570 recombinase A (recA) gene, partial cds

product length = 57

Forward primer 1 AAGATCACCAGTGCGCTCAA 20

Template 388 .................... 407

Reverse primer 1 GAGCTGGTTGATGAAGATCGC 21

Template 444 ..................... 424

*OTHER RESULTS WERE DELETED DUE TO MISALIGNMENT OF ONE OR MORE NUCLEOTIDES WITH THE SEQUENCE.
