# Supplementary material for: Investigating Useful Properties of Four Streptomyces Strains Active against Fusarium graminearum Growth and Deoxynivalenol Production on Wheat Grains by qPCR
Source: Toxins (Basel). 2020 Aug 31;12(9):560. doi: 10.3390/toxins12090560 (PMC7551252; doi:10.3390/toxins12090560)
Supplement: Supplementary file 1 [file toxins-12-00560-s001.zip › toxins-877381-Supplementary files/File S2.docx]

**PRODUCTS ON TARGET TEMPLATES (TRI12QF / TRI12DR) USING NCBI PRIMER BLAST (DEFAULT PARAMETERS, NR DATABASE)**

>[MH514957.1](https://www.ncbi.nlm.nih.gov/entrez/viewer.fcgi?db=nucleotide&id=1461845934) Fusarium graminearum isolate 04501 Tri core gene cluster, complete sequence

product length = 84

Forward primer 1 ATCTCAGCCAGACGACAGGT 20

Template 22849 .................... 22830

Reverse primer 1 CGAGGCGAGGTGTAATATCC 20

Template 22766 .................... 22785

>[MH514956.1](https://www.ncbi.nlm.nih.gov/entrez/viewer.fcgi?db=nucleotide&id=1461845923) Fusarium graminearum isolate N2-1 Tri core gene cluster, complete sequence

product length = 84

Forward primer 1 ATCTCAGCCAGACGACAGGT 20

Template 22849 .................... 22830

Reverse primer 1 CGAGGCGAGGTGTAATATCC 20

Template 22766 .................... 22785

>[MH514955.1](https://www.ncbi.nlm.nih.gov/entrez/viewer.fcgi?db=nucleotide&id=1461845912) Fusarium graminearum isolate 09-5a Tri core gene cluster, complete sequence

product length = 84

Forward primer 1 ATCTCAGCCAGACGACAGGT 20

Template 23435 .................... 23416

Reverse primer 1 CGAGGCGAGGTGTAATATCC 20

Template 23352 .................... 23371

>[MH514954.1](https://www.ncbi.nlm.nih.gov/entrez/viewer.fcgi?db=nucleotide&id=1461845901) Fusarium graminearum isolate N6-2 Tri core gene cluster, complete sequence

product length = 84

Forward primer 1 ATCTCAGCCAGACGACAGGT 20

Template 22877 .................... 22858

Reverse primer 1 CGAGGCGAGGTGTAATATCC 20

Template 22794 .................... 22813

>[MH514953.1](https://www.ncbi.nlm.nih.gov/entrez/viewer.fcgi?db=nucleotide&id=1461845890) Fusarium graminearum isolate 630 Tri core gene cluster, complete sequence

product length = 84

Forward primer 1 ATCTCAGCCAGACGACAGGT 20

Template 23391 .................... 23372

Reverse primer 1 CGAGGCGAGGTGTAATATCC 20

Template 23308 .................... 23327

>[MH514952.1](https://www.ncbi.nlm.nih.gov/entrez/viewer.fcgi?db=nucleotide&id=1461845879) Fusarium graminearum isolate N8-2 Tri core gene cluster, complete sequence

product length = 84

Forward primer 1 ATCTCAGCCAGACGACAGGT 20

Template 22856 .................... 22837

Reverse primer 1 CGAGGCGAGGTGTAATATCC 20

Template 22773 .................... 22792

>[MH514951.1](https://www.ncbi.nlm.nih.gov/entrez/viewer.fcgi?db=nucleotide&id=1461845868) Fusarium graminearum isolate 74b Tri core gene cluster, complete sequence

product length = 84

Forward primer 1 ATCTCAGCCAGACGACAGGT 20

Template 23454 .................... 23435

Reverse primer 1 CGAGGCGAGGTGTAATATCC 20

Template 23371 .................... 23390

>[MH514950.1](https://www.ncbi.nlm.nih.gov/entrez/viewer.fcgi?db=nucleotide&id=1461845857) Fusarium graminearum isolate N10-2 Tri core gene cluster, complete sequence

product length = 84

Forward primer 1 ATCTCAGCCAGACGACAGGT 20

Template 22806 .................... 22787

Reverse primer 1 CGAGGCGAGGTGTAATATCC 20

Template 22723 .................... 22742

>[MH514949.1](https://www.ncbi.nlm.nih.gov/entrez/viewer.fcgi?db=nucleotide&id=1461845846) Fusarium graminearum isolate N4-1 Tri core gene cluster, complete sequence

product length = 84

Forward primer 1 ATCTCAGCCAGACGACAGGT 20

Template 22835 .................... 22816

Reverse primer 1 CGAGGCGAGGTGTAATATCC 20

Template 22752 .................... 22771

>[MH514948.1](https://www.ncbi.nlm.nih.gov/entrez/viewer.fcgi?db=nucleotide&id=1461845835) Fusarium graminearum isolate N10-1 Tri core gene cluster, complete sequence

product length = 84

Forward primer 1 ATCTCAGCCAGACGACAGGT 20

Template 22849 .................... 22830

Reverse primer 1 CGAGGCGAGGTGTAATATCC 20

Template 22766 .................... 22785

>[MH514947.1](https://www.ncbi.nlm.nih.gov/entrez/viewer.fcgi?db=nucleotide&id=1461845824) Fusarium graminearum isolate N6-1 Tri core gene cluster, complete sequence

product length = 84

Forward primer 1 ATCTCAGCCAGACGACAGGT 20

Template 22870 .................... 22851

Reverse primer 1 CGAGGCGAGGTGTAATATCC 20

Template 22787 .................... 22806

>[MH514946.1](https://www.ncbi.nlm.nih.gov/entrez/viewer.fcgi?db=nucleotide&id=1461845813) Fusarium graminearum isolate 09-13a Tri core gene cluster, complete sequence

product length = 84

Forward primer 1 ATCTCAGCCAGACGACAGGT 20

Template 23423 .................... 23404

Reverse primer 1 CGAGGCGAGGTGTAATATCC 20

Template 23340 .................... 23359

>[MH514945.1](https://www.ncbi.nlm.nih.gov/entrez/viewer.fcgi?db=nucleotide&id=1461845802) Fusarium graminearum isolate Kr 275-1 Tri core gene cluster, complete sequence

product length = 84

Forward primer 1 ATCTCAGCCAGACGACAGGT 20

Template 22872 .................... 22853

Reverse primer 1 CGAGGCGAGGTGTAATATCC 20

Template 22789 .................... 22808

>[MH514944.1](https://www.ncbi.nlm.nih.gov/entrez/viewer.fcgi?db=nucleotide&id=1461845791) Fusarium graminearum isolate 237 Tri core gene cluster, complete sequence

product length = 84

Forward primer 1 ATCTCAGCCAGACGACAGGT 20

Template 23440 .................... 23421

Reverse primer 1 CGAGGCGAGGTGTAATATCC 20

Template 23357 .................... 23376

>[MH514943.1](https://www.ncbi.nlm.nih.gov/entrez/viewer.fcgi?db=nucleotide&id=1461845780) Fusarium graminearum isolate 16-43-tp Tri core gene cluster, complete sequence

product length = 84

Forward primer 1 ATCTCAGCCAGACGACAGGT 20

Template 23412 .................... 23393

Reverse primer 1 CGAGGCGAGGTGTAATATCC 20

Template 23329 .................... 23348

>[MH514942.1](https://www.ncbi.nlm.nih.gov/entrez/viewer.fcgi?db=nucleotide&id=1461845769) Fusarium graminearum isolate 114-2 Tri core gene cluster, complete sequence

product length = 84

Forward primer 1 ATCTCAGCCAGACGACAGGT 20

Template 22841 .................... 22822

Reverse primer 1 CGAGGCGAGGTGTAATATCC 20

Template 22758 .................... 22777

>[MH514941.1](https://www.ncbi.nlm.nih.gov/entrez/viewer.fcgi?db=nucleotide&id=1461845758) Fusarium graminearum isolate 09-03a Tri core gene cluster, complete sequence

product length = 84

Forward primer 1 ATCTCAGCCAGACGACAGGT 20

Template 23415 .................... 23396

Reverse primer 1 CGAGGCGAGGTGTAATATCC 20

Template 23332 .................... 23351

>[MH514940.1](https://www.ncbi.nlm.nih.gov/entrez/viewer.fcgi?db=nucleotide&id=1461845747) Fusarium graminearum isolate 23-4 Tri core gene cluster, complete sequence

product length = 84

Forward primer 1 ATCTCAGCCAGACGACAGGT 20

Template 23404 .................... 23385

Reverse primer 1 CGAGGCGAGGTGTAATATCC 20

Template 23321 .................... 23340

>[MH514939.1](https://www.ncbi.nlm.nih.gov/entrez/viewer.fcgi?db=nucleotide&id=1461845736) Fusarium graminearum isolate 433-2 Tri core gene cluster, complete sequence

product length = 84

Forward primer 1 ATCTCAGCCAGACGACAGGT 20

Template 22806 .................... 22787

Reverse primer 1 CGAGGCGAGGTGTAATATCC 20

Template 22723 .................... 22742

>[MH514938.1](https://www.ncbi.nlm.nih.gov/entrez/viewer.fcgi?db=nucleotide&id=1461845725) Fusarium graminearum isolate N5-1 Tri core gene cluster, complete sequence

product length = 84

Forward primer 1 ATCTCAGCCAGACGACAGGT 20

Template 22872 .................... 22853

Reverse primer 1 CGAGGCGAGGTGTAATATCC 20

Template 22789 .................... 22808

>[MH514937.1](https://www.ncbi.nlm.nih.gov/entrez/viewer.fcgi?db=nucleotide&id=1461845714) Fusarium graminearum isolate 37 Tri core gene cluster, complete sequence

product length = 84

Forward primer 1 ATCTCAGCCAGACGACAGGT 20

Template 23376 .................... 23357

Reverse primer 1 CGAGGCGAGGTGTAATATCC 20

Template 23293 .................... 23312

>[MH514936.1](https://www.ncbi.nlm.nih.gov/entrez/viewer.fcgi?db=nucleotide&id=1461845703) Fusarium graminearum isolate St-9 Tri core gene cluster, complete sequence

product length = 84

Forward primer 1 ATCTCAGCCAGACGACAGGT 20

Template 22843 .................... 22824

Reverse primer 1 CGAGGCGAGGTGTAATATCC 20

Template 22760 .................... 22779

>[MH514935.1](https://www.ncbi.nlm.nih.gov/entrez/viewer.fcgi?db=nucleotide&id=1461845692) Fusarium graminearum isolate N7-1 Tri core gene cluster, complete sequence

product length = 84

Forward primer 1 ATCTCAGCCAGACGACAGGT 20

Template 22877 .................... 22858

Reverse primer 1 CGAGGCGAGGTGTAATATCC 20

Template 22794 .................... 22813

>[MH514934.1](https://www.ncbi.nlm.nih.gov/entrez/viewer.fcgi?db=nucleotide&id=1461845681) Fusarium graminearum isolate 03132 Tri core gene cluster, complete sequence

product length = 84

Forward primer 1 ATCTCAGCCAGACGACAGGT 20

Template 22809 .................... 22790

Reverse primer 1 CGAGGCGAGGTGTAATATCC 20

Template 22726 .................... 22745

>[MH514933.1](https://www.ncbi.nlm.nih.gov/entrez/viewer.fcgi?db=nucleotide&id=1461845670) Fusarium graminearum isolate 04286 Tri core gene cluster, complete sequence

product length = 84

Forward primer 1 ATCTCAGCCAGACGACAGGT 20

Template 22840 .................... 22821

Reverse primer 1 CGAGGCGAGGTGTAATATCC 20

Template 22757 .................... 22776

>[MH514932.1](https://www.ncbi.nlm.nih.gov/entrez/viewer.fcgi?db=nucleotide&id=1461845659) Fusarium graminearum isolate 321 Tri core gene cluster, complete sequence

product length = 84

Forward primer 1 ATCTCAGCCAGACGACAGGT 20

Template 23411 .................... 23392

Reverse primer 1 CGAGGCGAGGTGTAATATCC 20

Template 23328 .................... 23347

>[MH514931.1](https://www.ncbi.nlm.nih.gov/entrez/viewer.fcgi?db=nucleotide&id=1461845648) Fusarium graminearum isolate 09-21a Tri core gene cluster, complete sequence

product length = 84

Forward primer 1 ATCTCAGCCAGACGACAGGT 20

Template 22877 .................... 22858

Reverse primer 1 CGAGGCGAGGTGTAATATCC 20

Template 22794 .................... 22813

>[MH514930.1](https://www.ncbi.nlm.nih.gov/entrez/viewer.fcgi?db=nucleotide&id=1461845637) Fusarium graminearum isolate 16-462-z Tri core gene cluster, complete sequence

product length = 84

Forward primer 1 ATCTCAGCCAGACGACAGGT 20

Template 23212 .................... 23193

Reverse primer 1 CGAGGCGAGGTGTAATATCC 20

Template 23129 .................... 23148

>[MH514929.1](https://www.ncbi.nlm.nih.gov/entrez/viewer.fcgi?db=nucleotide&id=1461845626) Fusarium graminearum isolate N4-2 Tri core gene cluster, complete sequence

product length = 84

Forward primer 1 ATCTCAGCCAGACGACAGGT 20

Template 22835 .................... 22816

Reverse primer 1 CGAGGCGAGGTGTAATATCC 20

Template 22752 .................... 22771

>[MH514928.1](https://www.ncbi.nlm.nih.gov/entrez/viewer.fcgi?db=nucleotide&id=1461845615) Fusarium graminearum isolate St-6 Tri core gene cluster, complete sequence

product length = 84

Forward primer 1 ATCTCAGCCAGACGACAGGT 20

Template 22863 .................... 22844

Reverse primer 1 CGAGGCGAGGTGTAATATCC 20

Template 22780 .................... 22799

>[MH514927.1](https://www.ncbi.nlm.nih.gov/entrez/viewer.fcgi?db=nucleotide&id=1461845604) Fusarium graminearum isolate 09-04a Tri core gene cluster, complete sequence

product length = 84

Forward primer 1 ATCTCAGCCAGACGACAGGT 20

Template 23415 .................... 23396

Reverse primer 1 CGAGGCGAGGTGTAATATCC 20

Template 23332 .................... 23351

>[MH514926.1](https://www.ncbi.nlm.nih.gov/entrez/viewer.fcgi?db=nucleotide&id=1461845593) Fusarium graminearum isolate 16-92-z Tri core gene cluster, complete sequence

product length = 84

Forward primer 1 ATCTCAGCCAGACGACAGGT 20

Template 23500 .................... 23481

Reverse primer 1 CGAGGCGAGGTGTAATATCC 20

Template 23417 .................... 23436

>[MH514925.1](https://www.ncbi.nlm.nih.gov/entrez/viewer.fcgi?db=nucleotide&id=1461845582) Fusarium graminearum isolate 16-21-z Tri core gene cluster, complete sequence

product length = 84

Forward primer 1 ATCTCAGCCAGACGACAGGT 20

Template 22649 .................... 22630

Reverse primer 1 CGAGGCGAGGTGTAATATCC 20

Template 22566 .................... 22585

>[MH514924.1](https://www.ncbi.nlm.nih.gov/entrez/viewer.fcgi?db=nucleotide&id=1461845571) Fusarium graminearum isolate 09-53b Tri core gene cluster, complete sequence

product length = 84

Forward primer 1 ATCTCAGCCAGACGACAGGT 20

Template 23418 .................... 23399

Reverse primer 1 CGAGGCGAGGTGTAATATCC 20

Template 23335 .................... 23354

>[LT598660.1](https://www.ncbi.nlm.nih.gov/nucleotide/1043015349?from=5609360&to=5609443&report=gbwithparts) Fusarium culmorum genome assembly, chromosome: II

product length = 84

Forward primer 1 ATCTCAGCCAGACGACAGGT 20

Template 5609443 .................... 5609424

Reverse primer 1 CGAGGCGAGGTGTAATATCC 20

Template 5609360 .................... 5609379

>[KU572434.1](https://www.ncbi.nlm.nih.gov/entrez/viewer.fcgi?db=nucleotide&id=1041495539) Fusarium aff. graminearum CBS 138562 trichothecene biosynthetic gene cluster, complete sequence

product length = 84

Forward primer 1 ATCTCAGCCAGACGACAGGT 20

Template 22167 .................... 22148

Reverse primer 1 CGAGGCGAGGTGTAATATCC 20

Template 22084 .................... 22103

>[KU572433.1](https://www.ncbi.nlm.nih.gov/entrez/viewer.fcgi?db=nucleotide&id=1041495528) Fusarium aff. graminearum CBS 119173 trichothecene biosynthetic gene cluster, complete sequence

product length = 84

Forward primer 1 ATCTCAGCCAGACGACAGGT 20

Template 22324 .................... 22305

Reverse primer 1 CGAGGCGAGGTGTAATATCC 20

Template 22241 .................... 22260

>[KU572432.1](https://www.ncbi.nlm.nih.gov/entrez/viewer.fcgi?db=nucleotide&id=1041495517) Fusarium aff. graminearum CBS 139513 trichothecene biosynthetic gene cluster, complete sequence

product length = 84

Forward primer 1 ATCTCAGCCAGACGACAGGT 20

Template 25902 .................... 25883

Reverse primer 1 CGAGGCGAGGTGTAATATCC 20

Template 25819 .................... 25838

>[KU572431.1](https://www.ncbi.nlm.nih.gov/entrez/viewer.fcgi?db=nucleotide&id=1041495506) Fusarium aff. graminearum CBS 139514 trichothecene biosynthetic gene cluster, complete sequence

product length = 84

Forward primer 1 ATCTCAGCCAGACGACAGGT 20

Template 25902 .................... 25883

Reverse primer 1 CGAGGCGAGGTGTAATATCC 20

Template 25819 .................... 25838

>[KU572429.1](https://www.ncbi.nlm.nih.gov/entrez/viewer.fcgi?db=nucleotide&id=1041495482) Fusarium aff. graminearum CBS 138561 trichothecene biosynthetic gene cluster, complete sequence

product length = 84

Forward primer 1 ATCTCAGCCAGACGACAGGT 20

Template 25709 .................... 25690

Reverse primer 1 CGAGGCGAGGTGTAATATCC 20

Template 25626 .................... 25645

>[KU572428.1](https://www.ncbi.nlm.nih.gov/entrez/viewer.fcgi?db=nucleotide&id=1041495471) Fusarium aff. graminearum CBS 104.09 trichothecene biosynthetic gene cluster, complete sequence

product length = 84

Forward primer 1 ATCTCAGCCAGACGACAGGT 20

Template 25900 .................... 25881

Reverse primer 1 CGAGGCGAGGTGTAATATCC 20

Template 25817 .................... 25836

>[KU572427.1](https://www.ncbi.nlm.nih.gov/entrez/viewer.fcgi?db=nucleotide&id=1041495460) Fusarium culmorum strain CBS 173.31 trichothecene biosynthetic gene cluster, complete sequence

product length = 84

Forward primer 1 ATCTCAGCCAGACGACAGGT 20

Template 23349 .................... 23330

Reverse primer 1 CGAGGCGAGGTGTAATATCC 20

Template 23266 .................... 23285

>[KU572426.1](https://www.ncbi.nlm.nih.gov/entrez/viewer.fcgi?db=nucleotide&id=1041495449) Fusarium culmorum strain MUCL53469 trichothecene biosynthetic gene cluster, complete sequence

product length = 84

Forward primer 1 ATCTCAGCCAGACGACAGGT 20

Template 23349 .................... 23330

Reverse primer 1 CGAGGCGAGGTGTAATATCC 20

Template 23266 .................... 23285

>[KU572424.1](https://www.ncbi.nlm.nih.gov/entrez/viewer.fcgi?db=nucleotide&id=1041495425) Fusarium culmorum strain CBS 110262 trichothecene biosynthetic gene cluster, complete sequence

product length = 84

Forward primer 1 ATCTCAGCCAGACGACAGGT 20

Template 23348 .................... 23329

Reverse primer 1 CGAGGCGAGGTGTAATATCC 20

Template 23265 .................... 23284

>[XM_011323866.1](https://www.ncbi.nlm.nih.gov/entrez/viewer.fcgi?db=nucleotide&id=758199604) Fusarium graminearum PH-1 hypothetical protein partial mRNA

product length = 84

Forward primer 1 ATCTCAGCCAGACGACAGGT 20

Template 39 .................... 58

Reverse primer 1 CGAGGCGAGGTGTAATATCC 20

Template 122 .................... 103

>[HG970333.1](https://www.ncbi.nlm.nih.gov/nucleotide/699037166?from=5415106&to=5415189&report=gbwithparts) Fusarium graminearum chromosome 2, complete genome

product length = 84

Features associated with this product:

[unnamed protein product](https://www.ncbi.nlm.nih.gov/nucleotide/699037166?from=5413261&to=5415227&report=gbwithparts)

Forward primer 1 ATCTCAGCCAGACGACAGGT 20

Template 5415189 .................... 5415170

Reverse primer 1 CGAGGCGAGGTGTAATATCC 20

Template 5415106 .................... 5415125

>[AF359361.3](https://www.ncbi.nlm.nih.gov/entrez/viewer.fcgi?db=nucleotide&id=28202133) Gibberella zeae strain GZ3639 trichothecene gene cluster, complete sequence

product length = 84

Forward primer 1 ATCTCAGCCAGACGACAGGT 20

Template 40282 .................... 40263

Reverse primer 1 CGAGGCGAGGTGTAATATCC 20

Template 40199 .................... 40218

>[AF336366.2](https://www.ncbi.nlm.nih.gov/entrez/viewer.fcgi?db=nucleotide&id=20475390) Gibberella zeae H-11 trichothecene biosynthesis gene cluster, complete sequence

product length = 84

Forward primer 1 ATCTCAGCCAGACGACAGGT 20

Template 22782 .................... 22763

Reverse primer 1 CGAGGCGAGGTGTAATATCC 20

Template 22699 .................... 22718

>[AY102605.1](https://www.ncbi.nlm.nih.gov/entrez/viewer.fcgi?db=nucleotide&id=21429592) Gibberella zeae strain NRRL 6394 trichothecene gene cluster, partial sequence

product length = 84

Forward primer 1 ATCTCAGCCAGACGACAGGT 20

Template 18534 .................... 18515

Reverse primer 1 CGAGGCGAGGTGTAATATCC 20

Template 18451 .................... 18470

>[AY102603.1](https://www.ncbi.nlm.nih.gov/entrez/viewer.fcgi?db=nucleotide&id=21429574) Gibberella zeae strain NRRL 5883 trichothecene gene cluster, partial sequence

product length = 84

Forward primer 1 ATCTCAGCCAGACGACAGGT 20

Template 18494 .................... 18475

Reverse primer 1 CGAGGCGAGGTGTAATATCC 20

Template 18411 .................... 18430

>[AY102602.1](https://www.ncbi.nlm.nih.gov/entrez/viewer.fcgi?db=nucleotide&id=21429565) Fusarium culmorum strain NRRL 3288 trichothecene gene cluster, partial sequence

product length = 84

Forward primer 1 ATCTCAGCCAGACGACAGGT 20

Template 18898 .................... 18879

Reverse primer 1 CGAGGCGAGGTGTAATATCC 20

Template 18815 .................... 18834

>[AY102599.1](https://www.ncbi.nlm.nih.gov/entrez/viewer.fcgi?db=nucleotide&id=21429538) Gibberella zeae strain NRRL 29169 trichothecene gene cluster, partial sequence

product length = 84

Forward primer 1 ATCTCAGCCAGACGACAGGT 20

Template 18495 .................... 18476

Reverse primer 1 CGAGGCGAGGTGTAATATCC 20

Template 18412 .................... 18431

>[AY102597.1](https://www.ncbi.nlm.nih.gov/entrez/viewer.fcgi?db=nucleotide&id=21429520) Fusarium boothii strain NRRL 29105 trichothecene gene cluster, partial sequence

product length = 84

Forward primer 1 ATCTCAGCCAGACGACAGGT 20

Template 18502 .................... 18483

Reverse primer 1 CGAGGCGAGGTGTAATATCC 20

Template 18419 .................... 18438

>[AY102595.1](https://www.ncbi.nlm.nih.gov/entrez/viewer.fcgi?db=nucleotide&id=21429502) Fusarium boothii strain NRRL 29020 trichothecene gene cluster, partial sequence

product length = 84

Forward primer 1 ATCTCAGCCAGACGACAGGT 20

Template 18509 .................... 18490

Reverse primer 1 CGAGGCGAGGTGTAATATCC 20

Template 18426 .................... 18445

>[AY102594.1](https://www.ncbi.nlm.nih.gov/entrez/viewer.fcgi?db=nucleotide&id=21429493) Fusarium boothii strain NRRL 29011 trichothecene gene cluster, partial sequence

product length = 84

Forward primer 1 ATCTCAGCCAGACGACAGGT 20

Template 18503 .................... 18484

Reverse primer 1 CGAGGCGAGGTGTAATATCC 20

Template 18420 .................... 18439

>[AY102584.1](https://www.ncbi.nlm.nih.gov/entrez/viewer.fcgi?db=nucleotide&id=21429403) Gibberella zeae strain NRRL 28336 trichothecene gene cluster, partial sequence

product length = 84

Forward primer 1 ATCTCAGCCAGACGACAGGT 20

Template 18363 .................... 18344

Reverse primer 1 CGAGGCGAGGTGTAATATCC 20

Template 18280 .................... 18299

>[AY102581.1](https://www.ncbi.nlm.nih.gov/entrez/viewer.fcgi?db=nucleotide&id=21429376) Gibberella zeae strain NRRL 28063 trichothecene gene cluster, partial sequence

product length = 84

Forward primer 1 ATCTCAGCCAGACGACAGGT 20

Template 18494 .................... 18475

Reverse primer 1 CGAGGCGAGGTGTAATATCC 20

Template 18411 .................... 18430

>[AY102579.1](https://www.ncbi.nlm.nih.gov/entrez/viewer.fcgi?db=nucleotide&id=21429358) Fusarium boothii strain NRRL 26916 trichothecene gene cluster, partial sequence

product length = 84

Forward primer 1 ATCTCAGCCAGACGACAGGT 20

Template 18506 .................... 18487

Reverse primer 1 CGAGGCGAGGTGTAATATCC 20

Template 18423 .................... 18442

>[AY102571.1](https://www.ncbi.nlm.nih.gov/entrez/viewer.fcgi?db=nucleotide&id=21429286) Fusarium culmorum strain NRRL 25475 trichothecene gene cluster, partial sequence

product length = 84

Forward primer 1 ATCTCAGCCAGACGACAGGT 20

Template 18899 .................... 18880

Reverse primer 1 CGAGGCGAGGTGTAATATCC 20

Template 18816 .................... 18835

*OTHER RESULTS WERE DELETED DUE TO MISALIGNMENT OF ONE OR MORE NUCLEOTIDES WITH THE SEQUENCE.
