# Supplementary material for: Investigating Useful Properties of Four Streptomyces Strains Active against Fusarium graminearum Growth and Deoxynivalenol Production on Wheat Grains by qPCR
Source: Toxins (Basel). 2020 Aug 31;12(9):560. doi: 10.3390/toxins12090560 (PMC7551252; doi:10.3390/toxins12090560)
Supplement: Supplementary file 1 [file toxins-12-00560-s001.zip › toxins-877381-Supplementary files/File S3/00File 3 legend.docx]

File 3 description

1. MIQE checklist with information on primer design, experimental procedures, data analysis;
2. *In silico* primer evaluation *recA*;
3. *In silico* primer evaluation *TRI12*;
4. qPCR data for *TRI12*, *recA* and wheat *EF1α* gene including standard curves and calculations to assess normalized abundance of *Streptomyces* and *Fusarium* (4 files: abundance estimation shows calculation for transformation of Ct values to number of DNA molecules, 3 excel files include data of quantification obtained for the 3 primer sets used in this work);
5. qPCR Raw data (ID numbers of plate positions are reported in the excel files (n. 4).
