# Supplementary material for: Investigating Useful Properties of Four Streptomyces Strains Active against Fusarium graminearum Growth and Deoxynivalenol Production on Wheat Grains by qPCR
Source: Toxins (Basel). 2020 Aug 31;12(9):560. doi: 10.3390/toxins12090560 (PMC7551252; doi:10.3390/toxins12090560)
Supplement: Supplementary file 1 [file toxins-12-00560-s001.zip › toxins-877381-Supplementary files/File S3/01MIQE_checklist.docx]

Miqe checklist

**Experimental design**

See paper, material and methods. Assays were carried out by the first author of the paper in the University of Milan Plant Pathology laboratory.

- *Number within each group*

Four biological replicates, three technical replicates for qPCR measurement

- *Sample description (extraction procedures…)*

See manuscript, materials and methods

**Sample**

See manuscript, materials and methods

**Nucleic acid extraction**

See manuscript, materials and methods

**qPCR target information**

See additional files, results from Netprimer software.

In order to normalize the quantification data with the amount of wheat DNA, primers based on plant EF1α gene was used (Nicolaisen et al., 2009).

- *Gene symbol*

Primers for *F. graminearum* quantification were designed on *TRI12*.

Primers for *Streptomyces* spp. quantification were designed on *recA.*

- *Sequence accession number*

*recA* genes obtained as described in Materials and methods were submitted in GenBank with accession numbesr:

BankIt2246844 DEF09 MN207071
BankIt2246844 DEF20 MN207072
BankIt2246844 DEF39 MN207073
BankIt2246844 DEF48 MN207074

Primers for *F. graminearum* quantification were designed based on *TRI12* gene sequence NC_026475

- *Location of amplicon*

Primers based on *recA* are positioned between the positions 426 to 482 of sequence MN207071

Primers based on *TRI12* are positioned between the positions 39 to 122 of sequence NC_026475

- *Amplicon length*

Product size for primers based on *recA* gene is 57 bp and on *TRI12* 84 bp

- *In silico* specificity screen

See additional file of results from NCBI Primer blast File S1, File S2

- *Sequence alignment*

Figure S4

**qPCR oligonucleotides**

- *Primers sequence*

See manuscript, materials and methods

- *Manufacturer of oligonucleotides*

Eurofins Genomics, Ebersberg, Germany

- *Purification method*

**HPSF - reverse phase cartridge purification method (High Purity Salt Free).**

**qPCR protocol**

See manuscript, materials and methods

**qPCR validation**

Optimization of primers based on *recA* gene is available in 05.raw data_plate folder, subfolder “Primer recA optimization”

Melt curve data, NTCs, Cq as well as PCR efficiency calculation and calibration curves are supplied as additional files in folder “05.raw data_plate” and “04.Target quantification”.

Standard curves for each plate are available in folder “04.Target quantification”.

**Data analysis**

- *Normalization method*

Raw data and files for each target quantification with calculation and standard curves comparison for all the plates run within this study are available in folder “05.raw data_plate” and “04.Target quantification”.

Quality assessments were carried out using software QuantStudio™ Design & Analysis Software version 1.5.0 (Thermo Fisher Scientific, USA). Cq threshold was fixed manually and raw data were exported in Excel 2016 format. Data from standard curves of each plate were selected in order to perform the standard curves comparisons. Linear regression, slope and PCR efficiency was then calculated manually using excel functions. The standard curve with best PCR efficiency was used to calculate a correction factor to normalize the cq values of all the other plates.

Correction factor: Slope value of the best standard curve/Slope value of the standard curve

The correction factor was then multiplied to the Cq values of raw data and the amount of each target could be obtained. The equation of the best standard curve was used to calculate the final amount of each target for each samples.

Amount of each target: EXP((Cq-q)/m) were q is the y-intercept and m is the slope.

Technical replicates with a standard deviation higher than 0.2 were discarded. The means of technical replicates in ng for each sample were obtained and the copy numbers for each sample were calculated using the following formula.


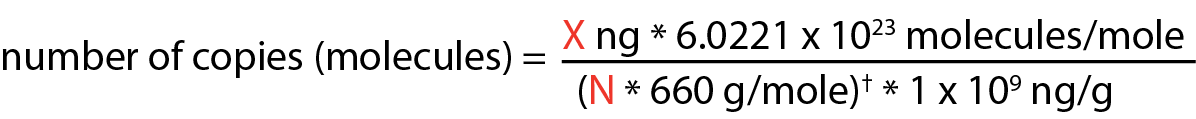


X= amount of the target in ng

N= length of the genome in bp

For *F. graminearum* N=36100000 bp

For streptomycetes N=9000000 bp

For wheat N=17000000000 bp (number of DNA molecules of wheat were divided by 6 as wheat is hexaploid)

Numbers of DNA molecules were used for statistical analyses described in the paper.
