# Supplementary material for: Investigating Useful Properties of Four Streptomyces Strains Active against Fusarium graminearum Growth and Deoxynivalenol Production on Wheat Grains by qPCR
Source: Toxins (Basel). 2020 Aug 31;12(9):560. doi: 10.3390/toxins12090560 (PMC7551252; doi:10.3390/toxins12090560)
Supplement: Supplementary file 1 [file toxins-12-00560-s001.zip › toxins-877381-Supplementary files/File S3/02NetPrimer_recA.pdf]

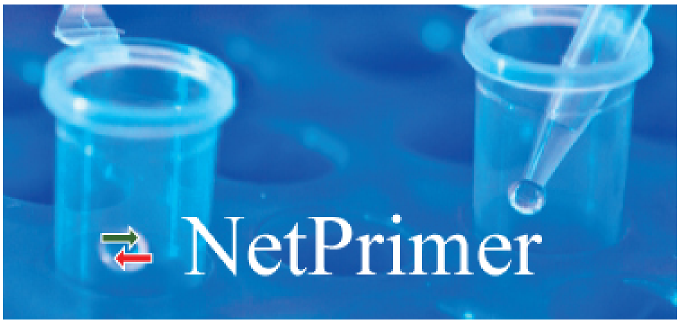

Free web based tool to analyze primers

PREMIER Biosoft

Oligo Analysis

Name	:	
Description	:	

Sequence #1 (5' to 3')

Length	:	20
AAGATCACCAGTGCGCTCAA		

Sequence #2 (5' to 3')

Length	:	21
GAGCTGGTTGATGAAGATCGC		

Reaction Conditions

Oligo Concentration	:	250.0	pM
Monovalent Ion Concentration	:	50.0	mM
Free Mg++ Ion Concentration	:	1.5	mM
Total Na[+] Equivalent	:	204.92	mM
Temperature for Free Energy Calculation	:	25.0	°C

Default Analyze

Analysis Results #1: AAGATCACCAGTGCGCTCAA

Rating	:	82.0	
Molecular Wt	:	6095.07	
Tm	:	58.87	°C
GC%	:	50.0	
GC Clamp	:	1	
nmol/A_{260}	:	5.04	
ug/A_{260}	:	30.71	
ΔG	:	-33.84	kcal/mol

3' end stability	:	-7.07	kcal/mol
ΔH	:	-148.3	kcal/mol
ΔS	:	-0.38	kcal/°K/mol
5' end ΔG	:	-6.59	kcal/mol
Self Dimer (ΔG)	:	-9.89	kcal/mol
Hairpin (ΔG)	:		kcal/mol
Repeats (# of pairs)	:		kcal/mol
Run (# of bases)	:		kcal/mol

Analysis Results #2: GAGCTGGTTGATGAAGATCGC

Rating	:	88.0	
Molecular Wt	:	6526.32	
Tm	:	59.69	°C
GC%	:	52.38	
GC Clamp	:	3	
nmol/A_{260}	:	4.81	
ug/A_{260}	:	31.38	
ΔG	:	-35.17	kcal/mol

3' end stability	:	-9.8	kcal/mol
ΔH	:	-155.8	kcal/mol
ΔS	:	-0.4	kcal/°K/mol
5' end ΔG	:	-7.91	kcal/mol
Self Dimer (ΔG)	:	-6.34	kcal/mol
Hairpin (ΔG)	:		kcal/mol
Repeats (# of pairs)	:		kcal/mol
Run (# of bases)	:		kcal/mol

Cross Dimer (ΔG)	:	-7.96	kcal/mol

Print

Copyright ©2019 PREMIER Biosoft. All rights reserved.
