# Supplementary material for: Investigating Useful Properties of Four Streptomyces Strains Active against Fusarium graminearum Growth and Deoxynivalenol Production on Wheat Grains by qPCR
Source: Toxins (Basel). 2020 Aug 31;12(9):560. doi: 10.3390/toxins12090560 (PMC7551252; doi:10.3390/toxins12090560)
Supplement: Supplementary file 1 [file toxins-12-00560-s001.zip › toxins-877381-Supplementary files/File S3/03NetPrimer _TRI12.pdf]

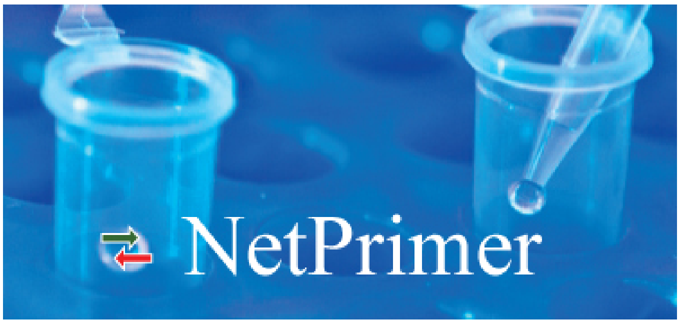

Free web based tool to analyze primers

PREMIER Biosoft

Oligo Analysis

Name	:	
Description	:	

Sequence #1 (5' to 3')

Length	:	20
ATCTCAGCCAGACGACAGGT		

Sequence #2 (5' to 3')

Length	:	20
CGAGGCGAGGTGTAATATCC		

Reaction Conditions

Oligo Concentration	:	250.0	pM
Monovalent Ion Concentration	:	50.0	mM
Free Mg++ Ion Concentration	:	1.5	mM
Total Na[+] Equivalent	:	204.92	mM
Temperature for Free Energy Calculation	:	25.0	°C

Default Analyze

Analysis Results #1: ATCTCAGCCAGACGACAGGT

Rating	:	100.0	
Molecular Wt	:	6111.07	
Tm	:	56.58	°C
GC%	:	55.0	
GC Clamp	:	2	
nmol/A_{260}	:	5.07	
ug/A_{260}	:	30.97	
ΔG	:	-32.44	kcal/mol

3' end stability	:	-7.96	kcal/mol
ΔH	:	-144.1	kcal/mol
ΔS	:	-0.37	kcal/°K/mol
5' end ΔG	:	-6.22	kcal/mol
Self Dimer (ΔG)	:		kcal/mol
Hairpin (ΔG)	:		kcal/mol
Repeats (# of pairs)	:		kcal/mol
Run (# of bases)	:		kcal/mol

Analysis Results #2: CGAGGCGAGGTGTAATATCC

Rating	:	91.0	
Molecular Wt	:	6182.1	
Tm	:	57.31	°C
GC%	:	55.0	
GC Clamp	:	2	
nmol/A_{260}	:	5.01	
ug/A_{260}	:	30.96	
ΔG	:	-34.44	kcal/mol

3' end stability	:	-7.08	kcal/mol
ΔH	:	-157.4	kcal/mol
ΔS	:	-0.41	kcal/°K/mol
5' end ΔG	:	-9.85	kcal/mol
Self Dimer (ΔG)	:	-4.91	kcal/mol
Hairpin (ΔG)	:		kcal/mol
Repeats (# of pairs)	:		kcal/mol
Run (# of bases)	:		kcal/mol

Cross Dimer (ΔG)	:		kcal/mol

Print

Copyright ©2019 PREMIER Biosoft. All rights reserved.
