# Supplementary material for: Investigating Useful Properties of Four Streptomyces Strains Active against Fusarium graminearum Growth and Deoxynivalenol Production on Wheat Grains by qPCR
Source: Toxins (Basel). 2020 Aug 31;12(9):560. doi: 10.3390/toxins12090560 (PMC7551252; doi:10.3390/toxins12090560)
Supplement: Supplementary file 1 [file toxins-12-00560-s001.zip › toxins-877381-Supplementary files/supple Figures S1,S2 and Table S1,S2.pdf]

Supplementary Materials: Investigating Useful Properties of Four *Streptomyces* Strains Active Against *Fusarium graminearum* Growth and Deoxynivalenol Production on Wheat Grains by qPCR

Elena Maria Colombo, Andrea Kunova, Claudio Gardana, Cristina Pizzatti, Paolo Simonetti, Paolo Cortesi, Marco Saracchi and Matias Pasquali

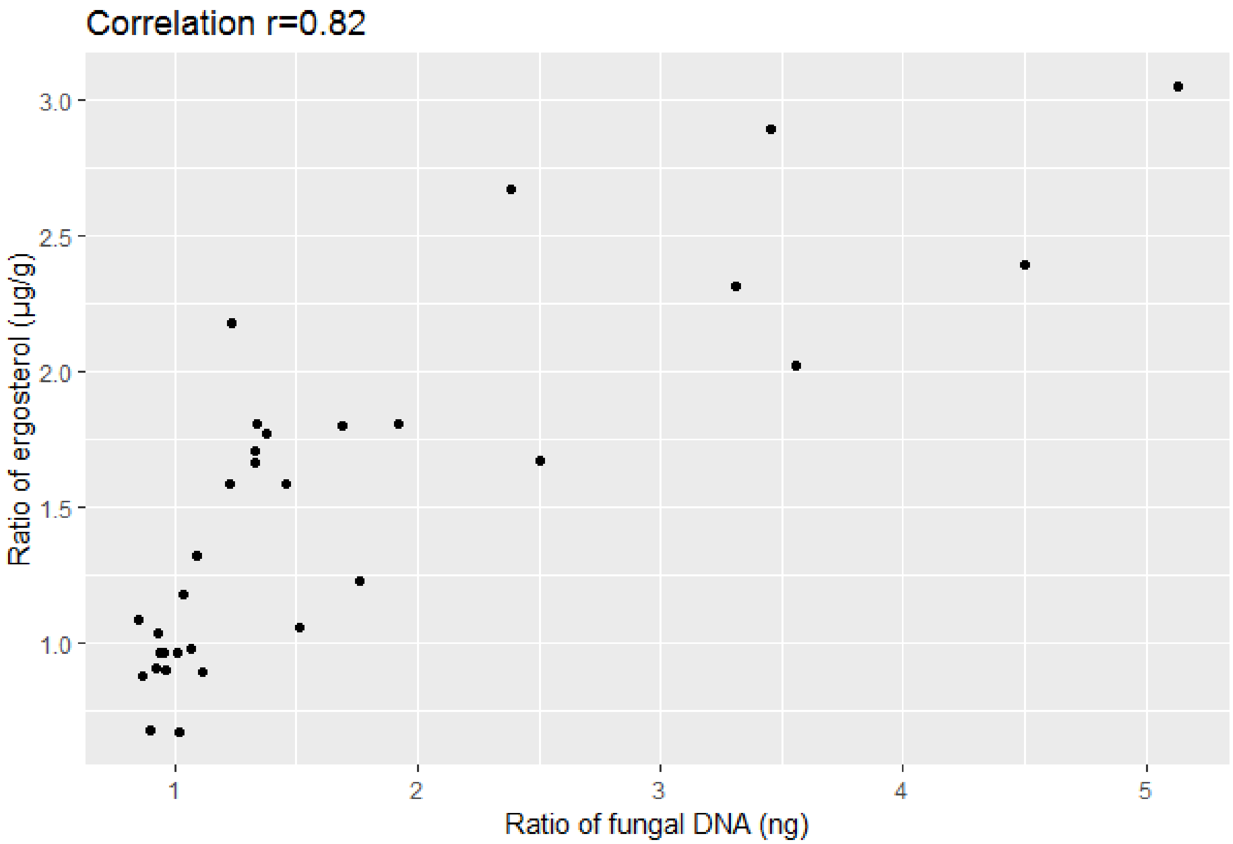

Figure S1. Scatter plot of ratios calculated between total ng of fungal DNA in control and treated samples and ratios of ergosterol (µg/g) in control and treated samples.

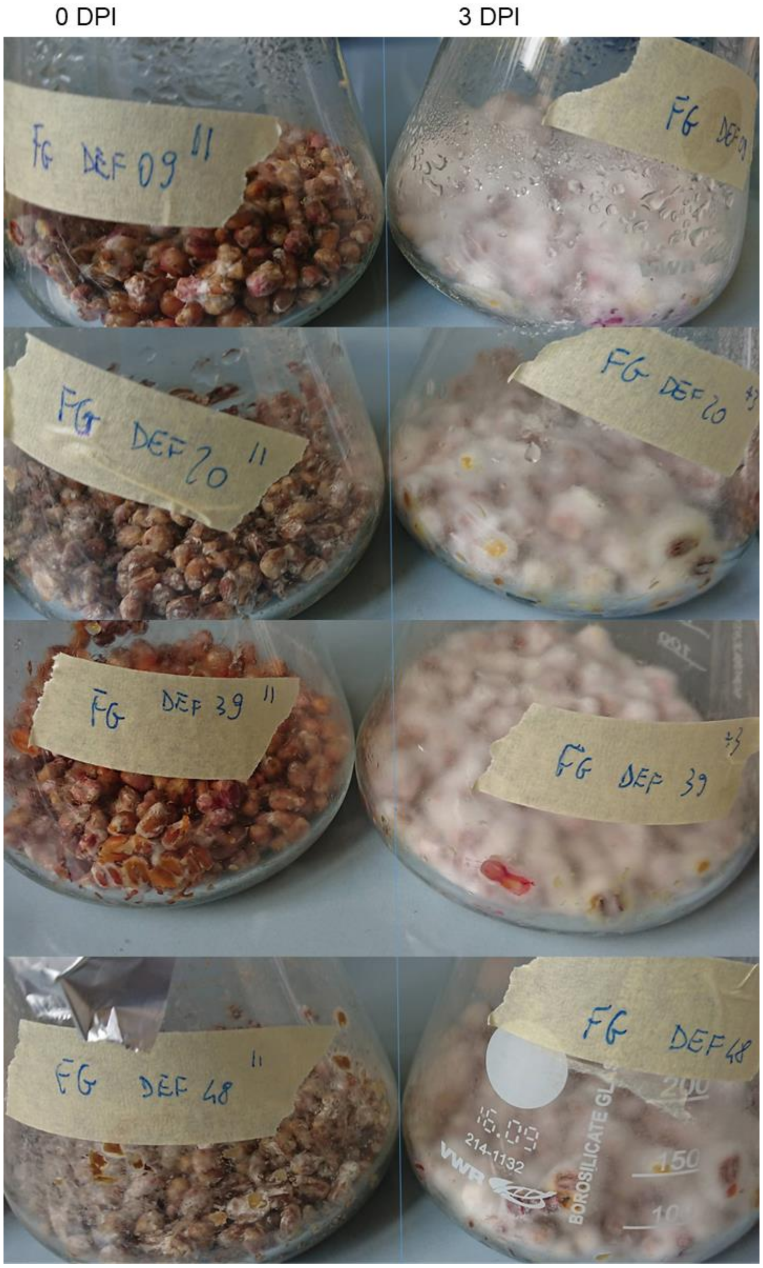

Figure S2. *Fusarium graminearum* growth in the flasks on wheat grains after co-inoculation (0 DPI) and late inoculation (3 DPI) with *Streptomyces* spp. strains DEF09, DEF20, DEF39 and DEF 48 after 11 days of incubation.

Table S1. *TRI12* and ergosterol quantification in blank samples (no-*Fusarium* or *Streptomyces* inoculation).

**Name of Blank Samples**	***TRI12* (ng)**	**Ergosterol (µg/g)**
Q32	0.02	0.00
Q49	Undetermined	0.00
Q74	Undetermined	0.00
Q105	Undetermined	0.00

Table S2. *P*-values of ANOVA analyses, comparing the effects of BCA treatments on fungal biomass detection by the two analysis methods (ergosterol and qPCR). In addition, results from Tukey's HSD post hoc comparison are listed as significant difference ($P < 0.05$) of the amount of ergosterol or fungal abundance in comparison with untreated control (no-*Streptomyces* inoculation).

***Streptomyces* Strain**	**Fungal Treatment**	**Time of BCA Inoculation**	***P*-value ANOVA Ergosterol**	**Tukey's HSD test (Ergosterol)**	***P*-value ANOVA qPCR**	**Tukey's HSD Test (Abbundance)**
DEF09	CS3005	3 DPI	0.043*	=	0.29	=
DEF39	CS3005	3 DPI	0.043*	=	0.29	=
DEF20	CS3005	3 DPI	0.70	=	0.44	=
DEF48	CS3005	3 DPI	0.70	=	0.44	=
DEF09	CS3005	0 DPI	4.42e-06*	-	0.01*	-
DEF39	CS3005	0 DPI	4.42e-06*	-	0.01*	-
DEF20	CS3005	0 DPI	0.00*	-	0.01*	-
DEF48	CS3005	0 DPI	0.00*	-	0.01*	-

= amount equal to control samples; - amount decreased in comparison to control samples; *$p < 0.05$ is considered significant.
